# Pollutants Transformation During the Regeneration Process of Fluid Catalytic Cracking Catalysts

**DOI:** 10.1002/anie.202513628

**Published:** 2025-11-04

**Authors:** Jiawei Bian, Robin Vogel, Pengfei Tian, Shuang Yang, Bohan Wang, Hao Ling, Fuzhen Xuan, Feng Ju, Bert M. Weckhuysen

**Affiliations:** ^1^ State Key Laboratory of Chemical Engineering School of Chemical Engineering East China University of Science and Technology–Utrecht University Joint Research Center for Sustainable and Circular Chemistry and Chemical Engineering East China University of Science and Technology Shanghai 200237 China; ^2^ Inorganic Chemistry and Catalysis group, Institute for Sustainable and Circular Chemistry, East China University of Science and Technology–Utrecht University Joint Research Center for Sustainable and Circular Chemistry and Chemical Engineering Utrecht University Universiteitsweg 99 Utrecht 3584 CG The Netherlands; ^3^ School of Mechanical and Power Engineering East China University of Science and Technology–Utrecht University Joint Research Center for Sustainable and Circular Chemistry and Chemical Engineering East China University of Science and Technology Shanghai 200237 China

**Keywords:** Coke deposits, Flue gas pollutants, Fluid catalytic cracking, *Operando* spectroscopy, Transformation mechanism

## Abstract

Fluid catalytic cracking (FCC) is the major process for heavy oil conversion in current refineries and is explored for the intake of renewable feedstocks, like biomass and plastic waste. Due to coke deposition, FCC catalysts undergo continuous reaction‐regeneration cycles. However, many gas pollutants are generated in the FCC regeneration process, and their emission characteristics and formation mechanisms are poorly understood. Here, we conducted stack tests of three industrial FCC units to monitor pollutant emissions. The spent catalysts were characterized to identify the carbon deposits formed. We developed a method to correlate the decomposition of carbon deposits and the formation of gas pollutants in regeneration experiments using in situ Raman spectroscopy, *operando* FT‐IR spectroscopy, and online gas‐phase FT‐IR spectroscopy. The evolution of coke species is significantly influenced by the oxygen content of the regeneration gas, leading to differences in emission concentration and formation temperature of various gas pollutants. The experimental results are compared with density functional theory (DFT) calculations to explain the formation of the major gas pollutants. This work is expected to advance pollutant emission prediction and control in FCC regeneration, thereby laying the foundation of future work in which different fossil‐based and renewable feedstock compositions can be compared, including their effect on gas pollutant formation.

## Graphical abstract

Fluid catalytic cracking is the main pollutant source in crude oil refineries due to spent catalyst regeneration. In this work, flue gas pollutants are monitored and coke deposits are identified. *Operando* and in situ spectroscopy are developed to explore coke species evolution, coupled with online gas infrared spectroscopy to detect pollutant formation. Combined with theoretical calculations, the gradual transformation mechanism from coke toward pollutants genesis is revealed.

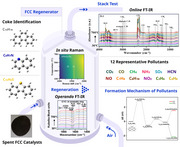

## Introduction

Fluid catalytic cracking (FCC) unit is at the core of the refining process, which convert heavy oil fractions into gasoline and liquefied gas with high value.^[^ ^1^, ^2^ ^]^ Moreover, an FCC unit is also a major air pollutant emission source due to the regeneration of spent catalysts, thereby leading not only to the formation of carbon dioxide, but also to different gas pollutants.^[^ ^3^, ^4^ ^]^ It is estimated that the FCC catalyst regenerator emissions account for more than 40% of the total pollutant emissions in refineries, which has attracted worldwide attention.^[^ ^5^, ^6^, ^7^, ^8^ ^]^ The U.S. Environmental Protection Agency (EPA) has listed hundreds of pollutants expected to be emitted by FCC units.^[^ ^9^ ^]^ Due to the complexity of the composition of FCC flue gas, emission characteristics are still not well understood. Therefore, the strategy for controlling the emission effectively is to understand the emission characteristics of FCC pollutants and the formation mechanism of FCC flue gas, which could present the transformation pathway from coke on the spent catalyst materials to gas pollutants during the regeneration process. Such information will also become important within the context of the gradually changing feedstocks as FCC units are also proposed to convert alternative feedstocks, such as agricultural and municipal waste, into valuable chemicals, such as light olefins and aromatics.^[^ ^10^ ^]^

The carbon deposits (further denoted as coke) that form on FCC materials due to deposition and polymerization of FCC feedstock, are the precursors of gas pollutants.^[^ ^11^, ^12^, ^13^ ^]^ Apart from the large hydrocarbons as the main components of coke deposits (e.g., naphthenes‐ and aromatics‐type molecules), hetero‐atom (e.g., N and S) compounds are non‐negligible. Nitrogen‐containing compounds, including N‐5, N‐6, and N‐Q with basicity, could cause catalyst deactivation by interacting with acid sites, while sulfur‐containing compounds could contribute to SO_2_ emissions.^[^ ^14^, ^15^ ^]^ Generally, the composition and structure of the coke deposits are broadly defined by various analytical techniques, including X‐ray photoelectron spectroscopy (XPS), nuclear magnetic resonance (NMR), elemental analysis (EA), confocal fluorescence microscopy (CFM), thermo‐gravimetrical analysis (TGA), temperature programmed desorption (TPD) in combination with mass spectrometry (MS) and gas chromatography (GC), infrared (IR) spectroscopy and Raman spectroscopy.^[^ ^2^, ^4^, ^12^, ^16^, ^17^, ^18^, ^19^ ^]^ However, the specific molecular structure of coke deposits remains unclear due to the interference of the zeolite material and other FCC components. Thus, the explicit characterization of coke deposits requires their complete separation from the catalyst material.^[^ ^16^ ^]^ The identified compounds can form the basis of studying the formation mechanism of FCC flue gas.

The focus of previous research has been on the evolution of coke deposits and the formation of gas pollutants. Hence, various analytical techniques, such as Fourier Transform‐Infrared Spectroscopy (FT‐IR) spectroscopy,^[^ ^20^, ^21^, ^22^ ^]^ Raman spectroscopy, ^[^ ^23^, ^24^, ^25^ ^]^ Ultraviolet‐Visible (UV–vis) spectroscopy,^[^ ^26^ ^]^ and Nuclear Magnetic Resonance (NMR), ^[^ ^27^ ^]^ have been used to study active intermediates under reaction conditions. Especially, Raman and FT‐IR spectroscopy are useful for following the formation and removal of coke inside the FCC materials,^[^ ^23^, ^28^, ^29^, ^30^ ^]^ the former method for the overall form of coke deposits and the latter method for a more detailed analysis of the functional groups of coke deposits. These spectroscopy techniques can also be used together to study the coke decomposition on the spent catalyst material coupled with the monitoring of gaseous product emission during the FCC regeneration process, revealing a clear and complete transfer mechanism. In addition to the regeneration experiment in the laboratory, theoretical calculations are also very useful for mechanistic studies, thereby making use of density functional theory (DFT) methods.^[^ ^23^, ^31^, ^32^ ^]^ Given the complex nature and related chemical composition of coke deposits and gas pollutants, DFT calculations can be very challenging, and relevant DFT studies on FCC regeneration processes are hence rare.

In this work, we have monitored three industrial FCC units to identify the main gas pollutants through the field test at the monitoring platform, which is located in the middle of the chimney stack, that is, a so‐called stack test^[^ ^7^, ^8^, ^9^ ^]^ has been performed. The spent catalyst materials collected from these commercial FCC units were analyzed and regenerated in the laboratory. The molecular structure of the coke deposits on the FCC materials was identified by EA, XPS, GC‐MS, TGA, and CFM. The regeneration experiments of spent FCC materials were carried out under different oxygen atmospheres (i.e., 0%, 10%, and 20% O_2_ in N_2_) to study the transformation mechanism. During the regeneration process, the decomposition behavior of the various coke species was recorded using in situ Raman and *operando* FT‐IR spectroscopy, and the formation of gas emissions was monitored by online gas‐phase FT‐IR spectroscopy. Besides, based on the coke molecules detected, DFT calculations were used to evaluate the evolution of the various active intermediates leading to gas pollutant formation. We identified a complete transfer path of FCC pollutants from coke deposits to flue gas, which could further help in developing FCC pollutant emission reduction strategies and improve FCC regeneration design options in the future, thereby laying the foundations for new research in which, e.g., municipal and agricultural waste (or their derived pyrolysis oils) are co‐processed with crude oil fractions, and hence making the refinery operations step‐by‐step more sustainable and ultimately also circular.

## Results and Discussion

### Stack Tests and Measurements of Greenhouse and Pollutant Gas Components

**Figure 1:**
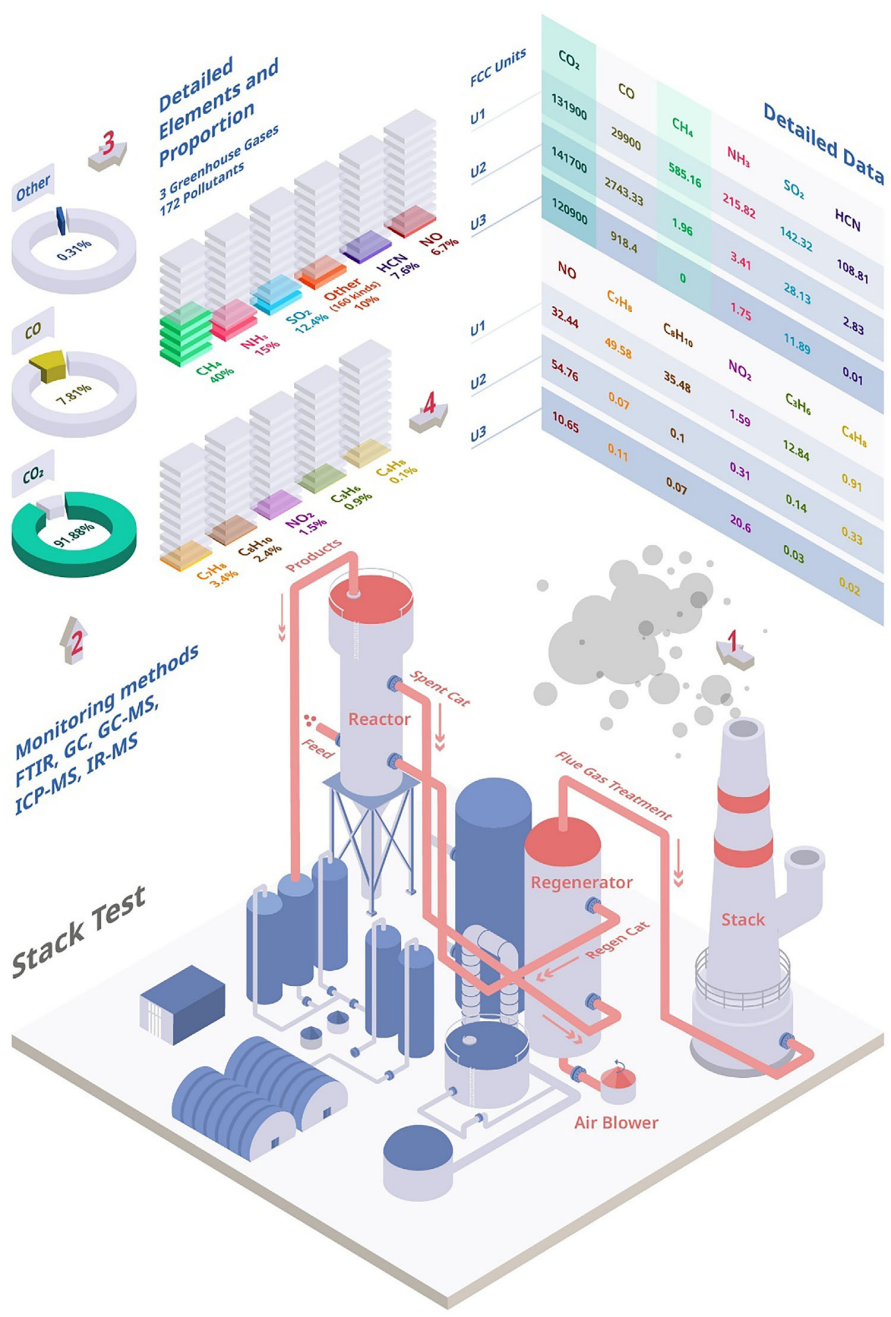
Monitoring results of stack tests in three industrial fluid catalytic cracking (FCC) units, labeled as U1, U2, and U3, thereby applying different regeneration methods, namely partial regeneration, partial regeneration with a CO boiler, and complete regeneration. a), Monitoring sites on the stack. b)–d), Monitoring methods and results of gas pollutants. CO_2_ and CO mainly consist of FCC pollutants in flue gas (b). The others are divided into 10 main gases and others (c). d) Detailed monitoring data for 12 representative gaseous products (ppm).

Stack tests were carried out on three industrial FCC units with different regeneration processes, including partial regeneration, partial regeneration with a CO boiler, and complete regeneration. The three FCC units cover the current two main regeneration types (i.e., complete regeneration and partial regeneration), which represent most regeneration processes in the refinery (Figure S1), thereby demonstrating the relevance of the detailed study conducted. The basic information of the three FCC units (further denoted as U1, U2, and U3) in China is shown in Table S1. After the tail treatment, the flue gas is emitted into the air through the stack, where the monitoring sites were set. 172 pollutant species and three greenhouse gas species (CO_2_, CH_4_, and N_2_O) were monitored by multiple methods (Figure 1 and Tables S2–S4).

Most pollutants are emitted at a considerable level from three FCC units, especially some with high concentrations, such as CO_2_, SO_2_, and NO*_x_*, and others with high toxicity, such as hydrogen cyanide (HCN) and polychlorinated dibenzodioxins (PCDDs), which suggests that the field monitoring pollutants need to be regulated. The emission concentrations of some pollutants (e.g., CO and NH_3_) are different among the three FCC units, which is mainly attributed to the differences in the regeneration process. The complexity of pollutant emission leads to great difficulties in comprehensive supervision since 172 pollutants and three greenhouse gases were detected. To simplify the problem, 10 pollutants and two greenhouse gases accounting for more than 90% of the total emissions are screened out as calibration (Figure 1), including CO_2_, CO, SO_2_, NO_2_, NO, NH_3_, HCN, CH_4_, propylene (C_3_H_6_), butene (C_4_H_8_), toluene (C_7_H_8_), and ethylbenzene (C_8_H_10_). The 12 representative gaseous products reflect the main emission characteristics of FCC regeneration and can be used to estimate the other pollutants by the correlation coefficient, referring to the emission inventories of EPA and European Environment Agency (EEA).^[^ ^9^, ^33^ ^]^ From the comprehensive monitoring data of three FCC units, the complex flue gas emissions are concentrated on the selected 10 pollutants and two greenhouse gases. Understanding the composition and structure of coke and its combustion mechanism provide new insights into the formation pathway of the 12 representative gaseous products.

### Coke Deposits Identification

**Figure 2:**
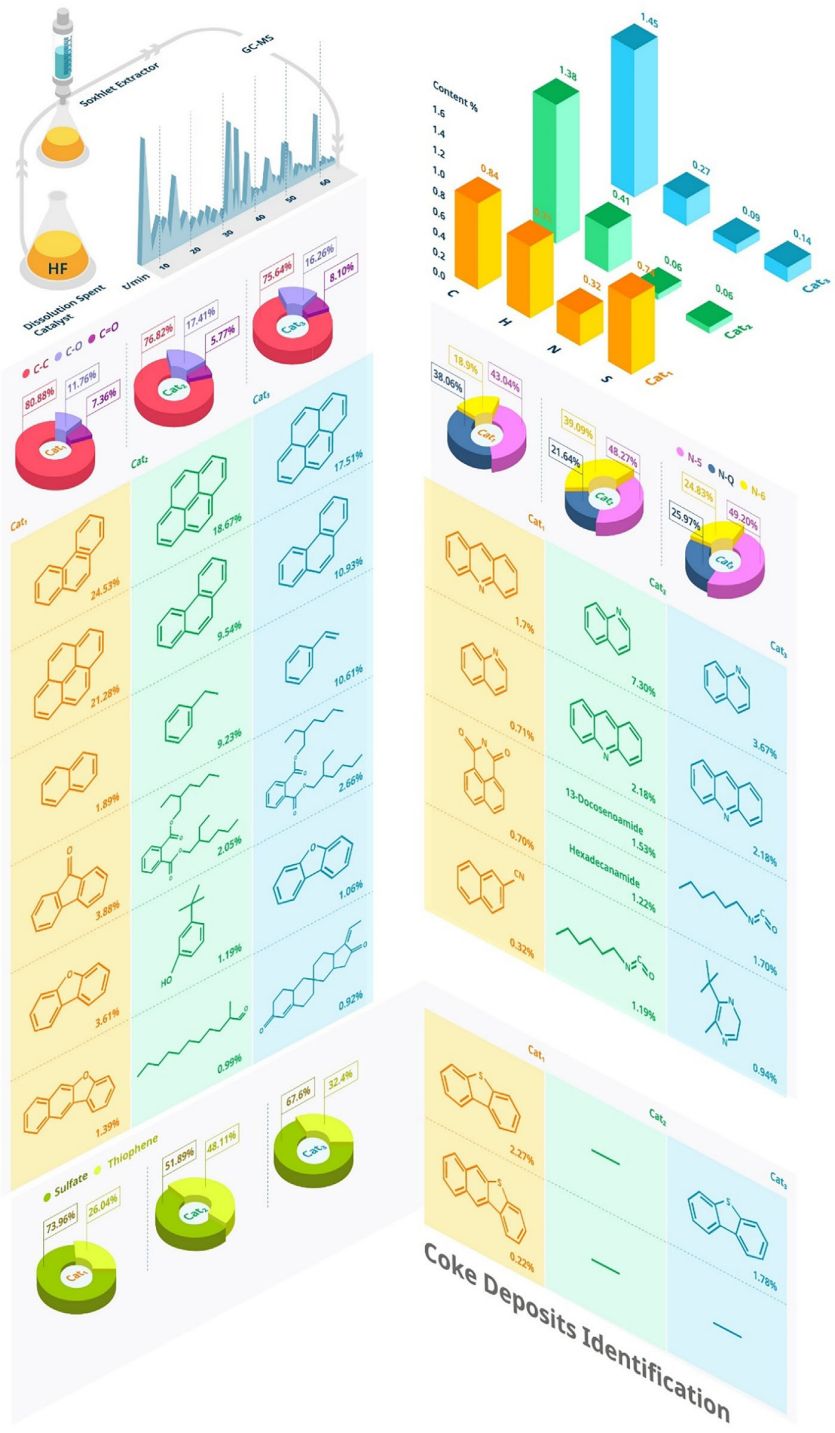
Composition of the coke deposits on the spent fluid catalytic cracking (FCC) materials. Elemental composition of coke from elemental analysis (EA) (Bar Charts). Species ratio of coke from X‐ray photoelectron spectroscopy (XPS) (Pie Charts). Molecular structure of coke from gas chromatography‐mass spectrometry (GC‐MS) after FCC materials dissolution and extraction (Tables).

Coke deposits, the precursors of the flue gas, are a complex mixture of multiple components that are challenging to identify. The identification of these species can provide insights into the mechanism of the formation of flue gas. Here, we use HF acid to dissolve the spent catalysts (Cat_1_, Cat_2_, and Cat_3_) to release the trapped coke and then characterize the extraction solution by GC‐MS to identify the detailed structure of the deposits. We find that the main compounds are phenanthrene and pyrene from the three studied catalysts, indicating that although the coke composition varies from sample to sample, the main compounds are similar (Figure 2 and Table S5). The coke content of the spent catalyst was analyzed using EA (Figure 2). The elemental composition of the spent catalysts varies strongly from sample to sample. The content of nitrogen and sulfur is much lower in Cat_2_ and Cat_3_ due to the feedstock hydrotreatment. XPS is used to identify the main element species. The same coke species are detected on the surface of the three spent catalysts (Figures 2 and S3), including three carbon species, three nitrogen species, and two sulfur species, which is consistent with the GC‐MS results.

The coke on the spent catalyst materials is mainly formed by hydrocarbon condensation reactions and hydrogen transfer reactions.^[^ ^34^ ^]^ Thus, the hydrocarbons are the main component of the coke, including a wide variety of (poly) aromatic hydrocarbons and a few aliphatic hydrocarbons. The aromatic hydrocarbons are polycyclic with a broad compositional distribution ranging from toluene to perylene while aliphatic hydrocarbons include a series of alkanes and a small number of alkenes and cycloalkanes (Table S5). Hydrocarbons are also recognized as C─C (aromatic and aliphatic carbon) at 284.8 eV in XPS (Figure S3).^[^ ^4^, ^19^ ^]^ The other two peaks in XPS C1s spectra belong to C─O and C═O species,^[^ ^4^ ^]^ which is consistent with the oxygen‐containing compounds detected by GC‐MS (Table S6). The oxygen‐containing compounds are divided into four categories: ketones, ethers, phenols, and esters. So far, little attention has been paid to the oxygen‐containing compounds. However, they actually play an important role in the regeneration process, due to the formation of CO/CO_2_ or oxygen‐containing radicals,^[^ ^35^ ^]^ which can react with nitrogen‐containing or sulfur‐containing compounds to generate air pollutants, such as NO*_x_* and SO_2_.

The emission of nitrogen species in the FCC unit has always been a concern. The nitrogen‐containing compounds in FCC feedstock, especially the nitrogen bases, easily poison the catalysts by interacting with the acid sites responsible for the cracking reaction.^[^ ^14^, ^36^ ^]^ The nitrogen species are converted to NO*_x_*, NH_3_, and HCN during the regeneration process, which is regulated as harmful. The main nitrogen‐containing compounds in coke are quinoline and acridine (Figure 2), corresponding to N‐6 (pyridine nitrogen) species in XPS spectra (Figure S3). Besides, other nitrogen species include carbazole, imidazole, aniline, nitrile, isocyanate, and amide (Table S7). Carbazole and imidazole are related to N‐5 (pyrrole nitrogen) species. Apart from this, other results of GC‐MS and XPS are inconsistent in types and ratios (Figure S3). The sulfur‐containing compounds detected by GC‐MS in the spent catalysts are dibenzothiophene and benzonaphthothiophene (Table S8), which are consistent with thiophenes as the main sulfur species in FCC feedstock.^[^ ^13^ ^]^ The sulfur‐containing compounds in Cat_2_ and Cat_3_ can rarely be detected due to feedstock hydrotreatments. The peaks of thiophene species are also observed in XPS data and the other peak is attributed to sulfate species (Figure S3). Generally, sulfate species are stable and difficult to decompose under regeneration conditions. During the regeneration process, thiophene species are broken down and oxidized into SO_2_.

From the characterization results, the coke composition is clearly displayed in the form of molecular structure. Based on these coke molecules, the formation of gas pollutants can be explained from an explicit starting point. However, the elucidation of the formation pathway of these pollutants is difficult due to the complexity of the precursors and the different regeneration processes, which have a great influence on the types and concentrations of pollutants. In addition to coke molecules, the intermediate regeneration process and final gas products also require clarification to describe the complete transfer mechanism.

### *Operando* Spectroscopy Results

**Figure 3:**
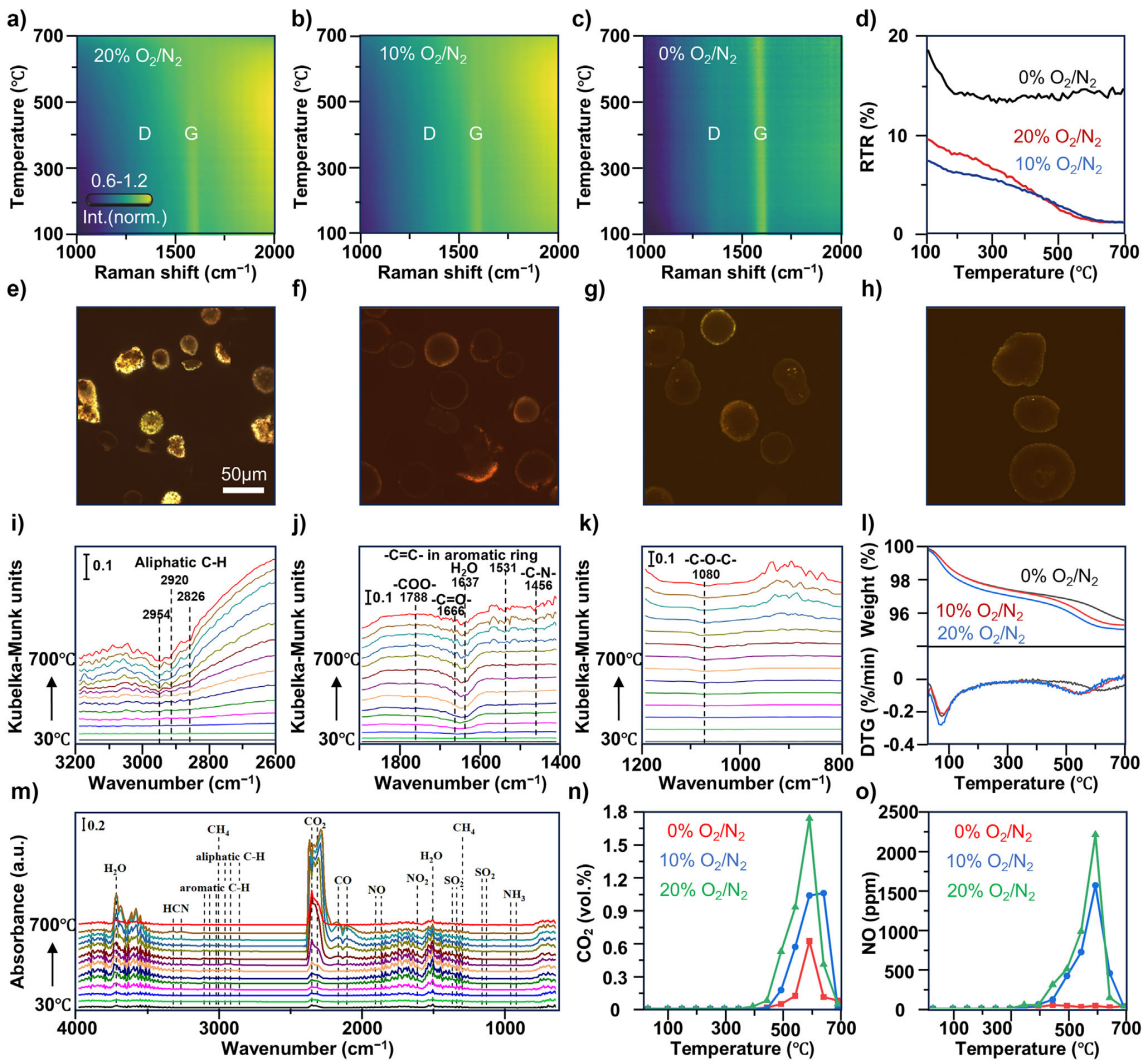
Evolution of coke deposits and gas pollutants during spent catalyst material (Cat_2_) regeneration. a)–c), Heatmap of Raman spectroscopy data in 20%, 10%, and 0% O_2_/N_2_. At each time, the spectrum is normalized to the maximum of the G‐band. d), Raman‐to‐total ratio (RTR) of the G‐band as a function of temperature for the regenerations in 20%, 10%, and 0% O_2_/N_2_. e)–h), Confocal fluorescence microscopy (CFM) images of spent and regenerated catalysts (0%, 10%, and 20% O_2_/N_2_). i)–k), *Operando* Fourier transform‐infrared (FT‐IR) spectra of Cat_2_ regeneration in 10% O_2_/N_2_, the difference spectra between the spectra at different temperatures and the spectrum at 30 °C. l), Thermogravimetric analysis (TGA) results of Cat_2_ in different atmospheres. m), online FT‐IR spectra of gas pollutants in 10% O_2_/N_2_ regeneration. n) and o), emission concentration of CO_2_ and NO.

*Operando* spectroscopy can be used to better understand the pathway of the deposition of coke into gas pollutants, although spectroscopy can be challenging under the harsh operating conditions. Here, we carry out the regeneration experiments on fixed beds in the laboratory from 30 °C to 700 °C under 0, 10%, and 20% O_2_/N_2_ atmosphere, which can present different regeneration processes. In situ Raman spectroscopy and *operando* FT‐IR spectroscopy were used to monitor the coke decomposition process, and online gas‐phase FT‐IR spectroscopy was used for quantitative analysis of gaseous products. Building the correlation between coke decomposition behavior and the corresponding content of the gas pollutants at the same regeneration conditions can help to provide valuable insights. We observed similar trends for the three FCC catalysts. But for the sake of brevity, only the results of Cat_2_ will be presented in Figure 3, while the data for Cat_1_ and Cat_3_ are illustrated in Figures S4–S15.

In situ Raman spectroscopy experiments were conducted to further elucidate the evolution of the coke species during the regeneration period. The related results are summarized in Figure 3a–d, as well as in Figure S4. The heatmaps of the normalized Raman spectra recorded on single catalyst particles of Cat_2_ show G‐ and D‐bands due to graphitic and disordered carbon, respectively,^[^ ^19^, ^37^, ^38^, ^39^, ^40^, ^41^ ^]^ together with intense background fluorescence due to electronic transitions in polyaromatic hydrocarbons. Background fluorescence is a common issue when performing Raman spectroscopy of zeolite‐based materials as it can obscure Raman features up to the point where they are no longer distinguishable.^[^ ^42^ ^]^ Therefore, we selected catalyst particles that exhibited well‐defined Raman features for the experiments. The heatmaps of the regenerations conducted in an oxygen‐containing atmosphere (Figure 3a,b) show that the D‐ and G‐bands gradually disappear, to become indistinguishable from the fluorescent background at about 500 °C. At this point, the fluorescence starts to dominate the spectra, indicating that graphitic carbon breaks down to form smaller fluorescent polyaromatic hydrocarbons. The spectra barely change in the absence of oxygen (Figure 3c), suggesting the prolonged presence of large graphitic structures under these conditions.

The evolution of the nature of graphitic carbon is typically assessed with the G‐ to D‐band intensity ratio.^[^ ^38^, ^39^, ^40^, ^41^ ^]^ A higher ratio indicates that the coke is composed of large and well‐ordered graphitic structures, whereas a lower ratio implies a composition of smaller defect‐rich graphitic patches. The intense fluorescence background made such an analysis for our samples rather complicated as the broad D‐band is hardly distinguishable from the fluorescent background. Therefore, we evaluated the degree of graphitization during the regeneration with the Raman‐to‐total ratio (RTR). The RTR is the ratio of the background‐corrected G‐band to the total signal at the wavelength of the G‐band (Figure S5).^[^ ^43^ ^]^ Tracking the RTR values during the regeneration process (Figure 3d) indicates a decreasing graphitization degree for the oxygen‐treated samples and the remaining presence of graphitic structures under nitrogen flow. These trends are reproduced for the other samples, in multiple experiments (Figure S6). The initial RTR varies from catalyst particle to catalyst particle due to the heterogeneity of the catalyst sample. Indeed, the reflection images of the samples show that the catalyst particles vary in size, shape, and color (Figure S7). The spectral fitting method allowed to determine the G‐ to D‐band intensity ratio too, but the data do not show clear trends (Figure S8).

In situ Raman spectroscopy results are also in line with CFM images (Figures 3e–h and S9). CFM measurements were conducted to present the type and spatial distribution of coke deposits on the catalyst materials.^[^ ^44^ ^]^ The longer the fluorescence wavelength, the higher the conjugation of coke species.^[^ ^45^ ^]^ The yellow, orange, and red fluorescence on the spent catalysts shows a mixture of different coke species with low and high poly‐aromaticity nature, corresponding to the D‐ and G‐ bands in the Raman spectra. After anaerobic regeneration of spent catalysts, except for the disappearance of yellow coke species with less conjugated nature,^[^ ^45^, ^46^ ^]^ the red fluorescence can still be observed on the surface of the regenerated catalyst, even a slight red shift compared with the spent catalyst. These coke species are more difficult to pyrolyze without oxygen and may be further converted into more conjugated poly‐aromatic compounds. When oxygen is introduced into the regeneration process, only the faint green fluorescence appears at the edge of the regenerated catalyst, indicating that oxygen helps poly‐aromatic compounds on the spent catalyst decompose into less conjugated coke species. These less conjugated coke species can be more easily removed from the catalyst, which is also proven by the lower coke removal onset temperature (500 °C) shown in the thermogravimetric analysis (TGA) plots (Figures 3l and S10).

*Operando* FT‐IR spectroscopy experiments were carried out to present the changes in functional groups of coke in the regeneration process. Actually, each FT‐IR spectrum is the difference spectrum obtained by subtracting the spectrum at 30 °C from the spectrum at different temperatures. The negative peaks suggest the breaking of the initial chemical bonds, and the bond assignments are summarized in Table S9. The main chemical bonds appearing in FT‐IR spectra are shown in Figures 3i–k and S11. Meanwhile, the emission behavior of gaseous products is confirmed by the online FT‐IR spectroscopy quantification of flue gas composition during the regeneration process (Figures 3m and S12), detailed infrared band analysis of main gases seen in Table S10.^[^ ^47^, ^48^, ^49^ ^]^ The concentration of gaseous products at different temperatures and oxygen concentrations is depicted in Figures 3n–o and S13–S15. In FT‐IR spectra, the peaks in the 3000–2800 cm^−1^ region are ascribed to stretching vibrations of aliphatic C─H species.^[^ ^50^ ^]^ These negative peaks, related to the alkanes in the coke, appear at 300 °C, indicating that these chained hydrocarbons easily decompose and volatilize. As for aromatic hydrocarbons with higher stability, aromatic ring C═C around 1600–1500 cm^−1 [^ ^51^ ^]^ begins to break at 400 °C, and a sharp decline can be seen after 550 °C.

The results are in line with the findings in the TGA measurements. In the gas online FT‐IR spectra, a series of vibrational peaks in the region 3200–2800 cm^−1^ are seen at 150–700 °C, belonging to VOCs and CH_4_. The VOCs mainly include propylene, butene, toluene, and ethylbenzene. The emission temperature region of propylene and butene is 300 °C to 700 °C. Toluene and ethylbenzene are released at 150–650 °C. The emission temperature of CH_4_ is much higher, around 550–700 °C. VOCs and CH_4_ are emitted in large quantities in the absence of oxygen. In 10% and 20% O_2_ atmosphere, a large amount of CO_2_ and CO is discharged at 400–700 °C, with the highest concentration point at 600 °C. These results confirm that the disappearance of coke signals from in situ Raman spectroscopy experiments corresponds to the removal of the coke species. Apart from alkanes and aromatic hydrocarbons, the oxygen‐containing compounds are also contributors to CO_2_ and CO emissions, especially in an oxygen‐free atmosphere. In the *operando* FT‐IR spectra, the negative peaks at around 1788, 1666, and 1080 cm^−1^ are ascribed to ─COO, C═O, and C─O─C.^[^ ^21^, ^49^, ^52^ ^]^ They are broken down at 350 °C, which can provide O atoms for regeneration.

The peak at ∼1456 cm^−1^ in the *operando* FT‐IR spectroscopy data is ascribed to the C─N bond,^[^ ^53^, ^54^ ^]^ which can be related to the presence of quinoline and carbazole species in coke. The C─N bond is broken down at 350 °C. The intensity of the negative peak is much lower at the beginning and becomes significantly higher after 550 °C. The C─N bond is located in the ring of quinoline and carbazole species, so its decomposition trend is consistent with that of the coke bulk in Raman spectra. Then N elements leave the coke and are converted to NO, NO_2_, HCN, and NH_3_, which is shown in the online gas FT‐IR spectroscopy data (Figure 3m). The emission temperature region of NO is 350 °C to 700 °C, and NO_2_ appears at 300 °C–500 °C and 600 °C–700 °C. The emission concentration of NO is much higher than that of NO_2_ in the presence of oxygen, which implies that NO can hardly be oxidized to NO_2_ in the FCC regeneration process. Usually, NH_3_ and HCN were considered intermediates of coke combustion and further oxidized to NO and NO_2_.^[^ ^14^, ^55^, ^56^ ^]^ But beyond expectation, NH_3_ and HCN are actually released at 550 °C–700 °C. Compared with stack test results, the emissions of NO and HCN in the experiments are quite higher. The discrepancies are attributed to flue gas treatment devices.

The emission characteristics of gas pollutants in the lab will also be a guide to the selection of flue gas treatment technology in the refinery. As for sulfur‐containing compounds, no relevant peak appears in the *operando* FT‐IR spectra. SO_2_ is the only sulfur‐containing gas detected in the online FT‐IR spectra. During the anaerobic regeneration of Cat_1_, the peak of SO_2_ with high intensity appears at 600–700 °C (Figure S12). Except for this experiment, the emission of SO_2_ has not been discovered in the other FT‐IR spectra.

### Evolution of Different Species During Catalyst Regeneration

Based on the combination of two spectroscopy methods and online FT‐IR spectroscopy, we present the evolution of the different species present in FCC materials during their regeneration. For carbon species, the evolution process is most significant throughout the entire regeneration process. When the temperature rises at the beginning, graphitic coke detected with Raman spectroscopy has not been decomposed. A small amount of toluene and ethylbenzene can be observed at 150–350 °C in all oxygen atmospheres applied, indicating that the simple aromatic hydrocarbons on the spent catalysts directly volatilize out at low‐temperature regions. After reaching the temperature of 300 °C, the decomposition of hydrocarbons leads to the formation of propylene and butene. The oxygen‐containing compounds (i.e., esters, ketones, and ethers) are broken down, and the O atom is exposed from ─COO, C═O, and C─O─C. Then, the graphitic structures break down into smaller polyaromatic hydrocarbons after reaching the temperature of 400 °C, as indicated by the decrease of the G‐band in the Raman spectra. In the oxygen‐free atmosphere, the smaller coke species directly enter the air to form VOCs (i.e., toluene and ethylbenzene). The exposed oxygen atoms may leave the coke due to the ring‐opening reaction. Therefore, a small quantity of CO_2_ and CO is emitted after 450 °C without O_2_. When the temperature reaches 500–550 °C, the graphitic structures fully break down into fluorescing polyaromatic hydrocarbons, as indicated by the disappearance of the G‐band in the Raman spectrum. The emission concentrations of toluene and ethylbenzene sharply decline. The other emission peak of propylene and butene at 600 °C can be seen in Figure S12. Meanwhile, CH_4_ is also emitted in large quantities. This can be explained by the hydrogen transfer process during coke pyrolysis, which is well‐studied during carbon‐pool mediated catalytic processes such as MTO, etc.^[^ ^23^, ^57^ ^]^ When oxygen is present, it can rapidly react with C or O free radicals after chemical bond breaking. The coke pieces are oxidized before they can escape from the catalysts. The emissions of VOCs and CH_4_ almost disappear, and only a small amount of VOCs is released in low‐temperature regions. A large amount of CO_2_ and CO are emitted after 400 °C. The emission temperature is 50 °C lower than that in the absence of oxygen. This is consistent with the coke decomposition process observed in the Raman spectra. Oxygen helps polyaromatics break down into less conjugated coke species, which are then rapidly oxidized to CO_2_ and CO. When the temperature continues to rise, especially around 550–600 °C, CO_2_ and CO are emitted at a super high level. The emission peak is in accordance with the coke removal onset temperature shown in the TGA, ascribed to the rapid oxidation of defect‐rich coke species.

The nitrogen‐containing compounds play an important role in the deactivation of FCC materials, so understanding nitrogen transfer is crucial for regeneration. From 350 °C to 550 °C, as graphitic coke is broken down, the ring of quinoline and carbazole species can be opened, exposing an N atom as a radical. In the absence of oxygen, most of N atoms still remain in the coke and NO emission concentration is close to 0. When oxygen is introduced, NO emission is sharply increased due to the reaction of O_2_ and N radicals. The peaks of NH_3_ and HCN in the FT‐IR spectra can be rarely seen during this temperature range. After reaching the temperature of 550 °C, the ring‐open quinoline and carbazole species are further cleaved and finally removed from the catalysts, which results in the emission of HCN and NH_3_. They can only leave the system after the coke species are finally broken down. NH_3_ appears in 0% O_2_ atmosphere, while HCN can be seen in the 10% and 20% O_2_ atmosphere. The emission difference indicates that N radicals at the end directly break off to form NH_3_ without oxygen while oxygen makes adjacent C─C bonds more likely to break so that the entire C─N fragment enters the air to form HCN.

The sulfur‐containing species on the spent FCC material include sulfates and thiophenes. Sulfates are generally decomposed at temperatures above 700 °C in the absence of oxygen and are much more difficult under aerobic conditions. Thiophenes are considered to be the source of SO_2_. The emission temperature of SO_2_ reflects the high stability of the C─S bond in the thiophene ring. SO_2_ is emitted after Raman features due to graphitic coke fully disappearing. In contrast to CO_2_ and NO, the emission of SO_2_ was significantly decreased under aerobic conditions. Considering that metal cations exist in the zeolite framework and that the oxidation of SO_2_ is easier than NH_3_ and HCN, we propose that SO_2_ might convert to sulfate by oxygen and remain on the catalyst surface, which is also a reasonable explanation for the sulfates on the spent catalyst detected by XPS. The accumulation of sulfates has an irreversible effect on the acidity of the catalyst in continuous circulation in the fluidized bed process, leading to a decline in catalyst stability and activity. The SO_2_ emission characteristics would partly explain why the regeneration process usually begins with an oxygen‐free pyrolysis step before regeneration with air in real industrial processes.

### Formation Mechanism of Pollutants

To achieve an in‐depth understanding of the regeneration process of FCC materials, DFT calculations were employed to investigate the formation mechanism of the specific pollutants in the absence and presence of O_2_. All calculations were performed using the Gaussian 09 program. The geometrical structures were optimized at the B3LYP/6‐31G* level, and single‐point energies for all the molecules were calculated at the B2PLYP/def2‐TZVP level on the basis of geometry optimizations. The optimized structures of reactants and critical intermediates are shown in Figures S16–S18.

Based on the experimental results, the dominant coke molecules on spent FCC materials are C_14_H_10_, C_12_H_8_S, and C_7_H_9_N. These molecules are considered as the starting points for the formation of pollutants, such as CO, CO_2_, SO_2_, NO, NO_2_, HCN, and NH_3_. The reaction routes for the generation of pollutants containing C, N, and S were proposed to proceed via radical mechanisms. In the presence of O_2_, coke molecules primarily react with radicals, such as O, OH, and OOH, to produce organic pollutants. On the other hand, for the oxygen‐free regeneration, the pollutants could be produced via intramolecular or intermolecular reactions. Since the ratio of oxygen‐containing molecules of the coke deposits on spent FCC materials is negligible compared with the other components, O_2_ should be the primary oxygen source for the oxidation reactions during the regeneration process. Accordingly, the oxygen‐containing pollutants, such as CO, CO_2_, SO_2_, NO, and NO_2_, are proposed to be produced in the presence of O_2_, while the other pollutants could be produced under oxygen‐free conditions.

**Figure 4:**
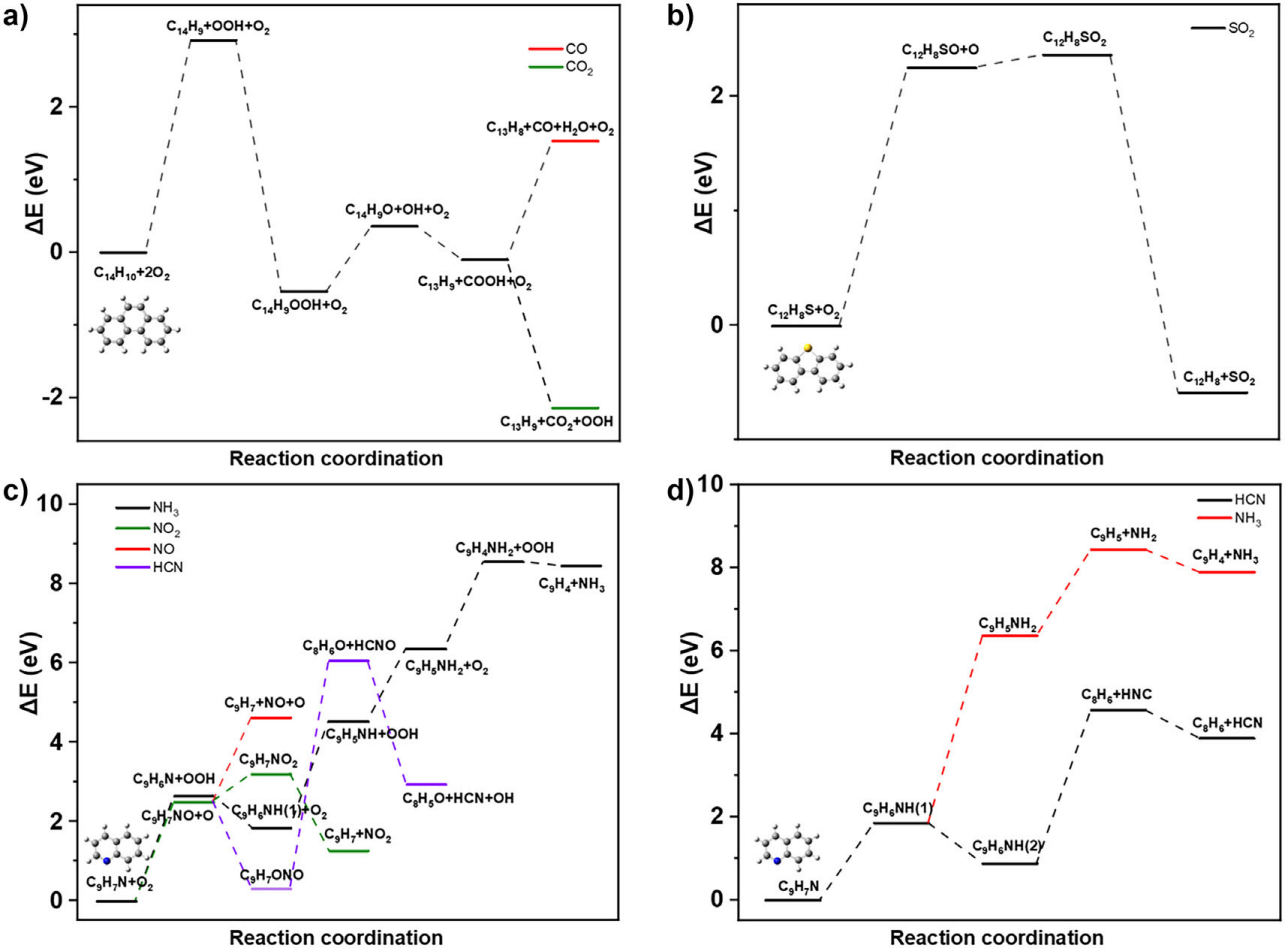
Density functional theory (DFT) calculated evolution path of coke molecules. a), Formation mechanism of CO and CO_2_ from C_14_H_10_ in the presence of O_2_. b), Formation mechanism of SO_2_ from C_12_H_8_S in the presence of O_2_. c), Formation mechanism of NO, NO_2_, NH_3_, and HCN from C_9_H_7_N in the presence of O_2_. d), Formation mechanism of NH_3_ and HCN from C_9_H_7_N under oxygen‐free conditions. Gray, blue, and white spheres represent C, N, and H atoms, respectively.

Hence, we have performed DFT calculations for the three model compounds in the absence and presence of O_2_. The results are summarized in Figure 4. As shown in Figure 4a, we took the oxidation of C_14_H_10_, which is the most abundant coke species, as the typical reaction for the production of CO and CO_2_. The results indicate that CO and CO_2_ are produced via the assistance of OOH and OH radicals. The highest reaction energy of the elementary steps for the removal of C_14_H_10_ is that of the formation of OOH radical (2.92 eV), and COOH intermediate is the critical intermediate for the production of CO and CO_2_. The reaction energy of the elementary step for the production of CO_2_ (‐2.14 eV) from COOH is markedly lower than that of CO (1.53 eV), suggesting that CO_2_ is the primary regeneration product for the carbon‐containing coke molecules in the presence of O_2_. Since carbon is the most abundant species of coke on the FCC materials, we can conclude that CO_2_ would be the predominant product of the regeneration process based on the theoretical results, which explains the highest ratio of CO_2_ in the results. For the sulfur‐containing coke molecules, C_12_H_8_S presented the highest ratio according to the GC‐MS test. Hence, we used the C_12_H_8_S molecule as the origin of the formation of sulfur‐containing pollutants. The reaction path for the production of SO_2_ from C_12_H_8_S consists of three steps, and the elementary step with the highest reaction energy (2.25 eV) is the production of C_12_H_8_SO intermediate (Figure 4b). The highest elementary reaction energy for the removal of C_12_H_8_S (2.25 eV) is lower than that of C_14_H_10_ (2.92 eV), suggesting that SO_2_ could be produced at a lower temperature. According to the results, NO, NO_2_, HCN, and NH_3_ were produced during the regeneration process both in the presence of O_2_ and under oxygen‐free conditions. In theoretical studies, C_9_H_7_N was obtained as the nitrogen‐source, since it is the most abundant nitrogen‐containing molecule of the coke deposits on the spent FCC materials. NO and NO_2_ can only be produced in the presence of O_2_, and the production of NO_2_ is thermodynamically more favorable than NO. The experimental results showed that NO presented a higher ratio than NO_2_, which might stem from the transformation of NO_2_ to NO at high temperature of the regeneration process. HCN and NH_3_ could be produced under conditions both in the presence of and without O_2_.

However, the formation energy of NH_3_ in the presence of O_2_ is markedly higher than those of NO and NO_2_. Hence, NH_3_ was primarily produced under oxygen‐free conditions in the experiment, while HCN could be produced both in the presence of and with O_2_. The production of HCN thus could be controlled using lower concentration of O_2_. These observations are summarized in Figure 4c,d. For the C_14_H10 and C_12_H_8_S molecules, no stable pollutant molecules could be produced through their direct pyrolysis reactions under oxygen‐free conditions, and H_2_ or H_2_S was not detected in the experiment. Thus, the anaerobic transformation processes for these molecules should not be predominant reaction routes and were not considered in DFT calculations.

To evaluate the entropy effect at high temperature, free energy corrections were applied to the evolution of coke molecules at 700 °C and 1 atm. As shown in Figure S19, the entropy contribution under these conditions primarily enhances catalytic activity, facilitating the formation of most products, while selectivity remains largely unchanged.

### Effect of Precursors on Different Catalyst Materials

The coke deposits and the regenerated flue gas show the same trends for the studied FCC materials. However, minor differences lead to discrepancies in the regeneration process, as shown in Figures 2 and 3. From the aspect of carbon species, Cat_1_ presents a lower C/H ratio, with fewer conjugated coke species. Therefore, the emission temperature of CO_2_, CO, and VOCs moves forward a little bit during Cat_1_ regeneration. As for nitrogen species, the nitrogen content in Cat_1_ is much higher, leading to the emission of HCN and NH_3_ in the low‐temperature region in the aerobic atmosphere (Figure S13). The emission of nitrogen‐containing gases under an oxidizing atmosphere is roughly split into two peaks: a main peak at around 600 °C and an earlier peak that appears at ∼ 400 °C. The former peak is the same as that in three spent FCC catalysts, related to the final coke removal step just like the 600 °C peaks for CO_2_ and CO. The latter low‐temperature peak implies the facilitated evolution of NO, HCN, and NH_3_ when the larger conjugated coke breaks down into smaller species in the presence of oxygen. The evolution of NH_3_ and HCN species (instead of NO*_x_* species only) can be explained by the kinetic stability of the two species in an environment lacking oxidizing catalysts. The appearance of the low‐temperature peak is due to the extremely high nitrogen content in Cat_1_. The extra N cannot be accommodated by the large conjugated coke system and can only be bound to the coke species with a weaker interaction. These species can be released and converted into N‐containing pollutants during the decomposition of the larger system into defect‐rich coke, which happens at a lower temperature with the help of oxygen. For sulfur species, the higher sulfur content of Cat_1_ results in the significant emission of SO_2_ after 600 °C in the oxygen‐free atmosphere.

At last, it can be concluded that the combination of *operando* spectroscopy methods coupled with online gas product analysis helps to reveal the regeneration process of FCC spent catalysts. Oxygen has a great impact on coke decomposition and gas pollutants emission. The regeneration types determine the combustion intensity of coke and the emission characteristics of the related gas pollutants. Despite the spent catalysts originated from three industrial FCC units, similar trends are observed during the regeneration experiments, which indicates that it is possible to construct a transformation pathway widely applicable to different regeneration processes in various FCC units. The mechanism indicates that the regeneration process can be optimized with a combination of anaerobic and aerobic regeneration steps. The first anaerobic process converts N species to NH_3_ instead of HCN and promotes the decomposition of sulfates. NH_3_ can be regarded as a reducing agent in selective catalytic reduction (SCR) for the FCC flue gas, and the removal of sulfate will mitigate the irreversible deactivation of the catalysts. This first anaerobic step might lead to more extensive graphitization of the coke, which can be burned off in the next oxygen‐enriched combustion. The second aerobic step of the regeneration process has a higher coke removal rate than one‐step complete regeneration. VOCs and other organic compounds are oxidized to CO_2_. Moreover, a CO boiler in the flue gas treatment devices is effective for the removal of CO and VOCs, or catalytic oxidation technology is recommended. Thus, large amounts of CO_2_ in flue gas should be captured or recycled for greenhouse gas emission reduction. Various pollutants from FCC regeneration will be monitored and controlled with stricter new emission standards in the future.

## Conclusion

Through the stack test of three industrial FCC units, the emissions of 172 pollutant species and three greenhouse gas species in the flue gas were monitored. The complex flue gas composition was categorized by taking 12 representative gaseous products, which were used as monitoring targets for laboratory regeneration experiments. The spent catalyst materials from the industrial FCC units were analyzed by multiple analytical techniques to identify the molecular structure of the coke deposits formed. The coke species on the three FCC materials are very similar and mainly composed of phenanthrene and pyrene. The hetero‐atom aromatic compounds in the coke deposits are more complex, mainly including dibenzofuran and fluorenone in the oxygen‐containing compounds, quinoline and acridine in the nitrogen‐containing compounds, and dibenzothiophene in the sulfur‐containing compounds. In situ Raman spectroscopy and *operando* infrared spectroscopy with online gas product analysis were used to investigate the pollutant transformation during the FCC regeneration process. Using these methods, the regeneration experiments of the spent catalyst materials in the laboratory have indicated that oxygen content has a great effect on the evolution of coke species and the formation of gas pollutants. Anaerobic regeneration can only partially remove coke species and some may even convert into more conjugated graphitic species. In the absence of oxygen, CH_4_, VOCs, and NH_3_ are the main gas products, while sulfate on the catalyst materials can be broken down to mitigate irreversible deactivation. The participation of oxygen reduces the decomposition temperature of coke species and greatly increases the emission of the oxidation products formed. In particular, HCN is significantly produced under aerobic conditions. The dominant coke molecules and the typical gas pollutants were used for theoretical calculations to investigate the formation of possible intermediates. Combined with the simulation results, a complete transfer path from coke species into gas pollutants during the FCC regeneration process was established. Based on this mechanism, we propose a strategy for pollutant emission prediction and control in the FCC regeneration process, which provides valuable guidance for FCC unit operations to minimize the emission of pollutants. Moreover, impurities, such as nitrogen and sulfur, are abundant in waste plastics and biomass, and their conversion is the key for the refinery of the future. The nitrogen and sulfur conversion mechanisms identified in this study provide general support for the resource utilization of waste plastics and biomass. Especially, this work lays down a solid foundation for future work on FCC regeneration processes with biomass and plastic waste feedstock as a viable alternative to fossil‐derived feedstock. Beyond FCC regeneration, the methodology and mechanistic insights from this study could benefit other industrial processes involving carbonaceous deposits and heteroatom transformations, such as dry methane reforming, aromatics conversion processes and ammonia power plants. The operando spectroscopic approaches and reaction pathways developed here may help to optimize reaction conditions and mitigate emissions in these systems, where nitrogen and sulfur management are critical for sustainability.

## Supporting Information

The authors have cited additional references within the Supporting Information.^[^ ^58^ ^]^

## Conflict of Interests

The authors declare no conflict of interest.

## Supporting information



Supporting Information

## Data Availability

The data that support the findings of this study are available from the corresponding author upon reasonable request.
