# Supplementary material for: Pollutants Transformation During the Regeneration Process of Fluid Catalytic Cracking Catalysts
Source: Angew Chem Int Ed Engl. 2025 Nov 4;64(52):e13628. doi: 10.1002/anie.202513628 (PMC12723451; doi:10.1002/anie.202513628)
Supplement: Supplementary file 1 — Supporting Information [file ANIE-64-e13628-s001.docx]

**Table of Contents**

[1. Details and Background on Experimental Methods and Procedures 2](#_Toc208428329)

[2. Supplementary Results and Discussion 8](#_Toc208428330)

[S1. Emission Concentrations of Pollutants and Greenhouse Gases in the Flue Gases of Industrial Fluid Catalytic Cracking Units 8](#_Toc208428331)

[S2. Coke Deposits on the Spent Catalyst Materials 14](#_Toc208428332)

[S3. *In situ* Raman Spectroscopy Results 19](#_Toc208428333)

[S4. Confocal Fluorescence Microscopy Images 22](#_Toc208428334)

[S5. Thermogravimetric Analysis Results 23](#_Toc208428335)

[S6. *Operando* Infrared Spectroscopy Results 24](#_Toc208428336)

[S7. Online Gas-Phase Infrared Spectroscopy Results 26](#_Toc208428337)

[S8. Density Functional Theory Calculations 31](#_Toc208428338)

[S9. References 33](#_Toc208428339)

1. Details and Background on Experimental Methods and Procedures

1. **Industrial Fluid Catalytic Cracking Units**

An industrial fluid catalytic cracking (FCC) unit mainly consists of a reactor and a regenerator, as shown in Figure S1. The pre-heated heavy oil feedstock is sent into the riser reactor and combined with the hot catalyst material from the regenerator. The heavy oil is cracked into gasoline and base chemicals, such as propylene, at 490-550℃. Coke is also deposited on the FCC materials during the cracking process. We have further called the related materials spent FCC materials. The spent FCC materials are transported into the regenerator to burn off the coke deposits using air at 660-750℃. After the catalytic activity recovers, the regenerated FCC materials are sent back to the riser reactor, where they are mixed with the pre-heated heavy oil feedstock. The FCC materials are continuously recycled in the riser and regeneration reactor. Each cycle is considered to take around 10 min, while the regenerated FCC material generally spends only 1-2 s in the riser reactor, and most of the remaining time the FCC material in the regenerator reactor. During the regeneration process, the coke combustion results in the emissions of various pollutants and greenhouse gases in the flue gas stream, such as CO_2_, NO*_x_* (e.g., NO), SO_2_, and CO, and particulate matter (PM). The flue gas is generally purified by selective catalytic reduction (SCR) and wet flue gas desulfurization (WFGD), which is finally emitted into the air according to national and regional emission standards, which are for example GB 31570-2015 Emission Standard of Pollutants for Petroleum Industry in China. The emissions of pollutants are significantly affected by the regeneration process, which is commonly divided into full regeneration and partial regeneration according to the oxygen concentration and CO content. In the complete regeneration, O_2_ is adequately supplied, and the CO content is usually less than 1%. In partial regeneration, O_2_ is limited, and the CO content can be between 3%-5%.


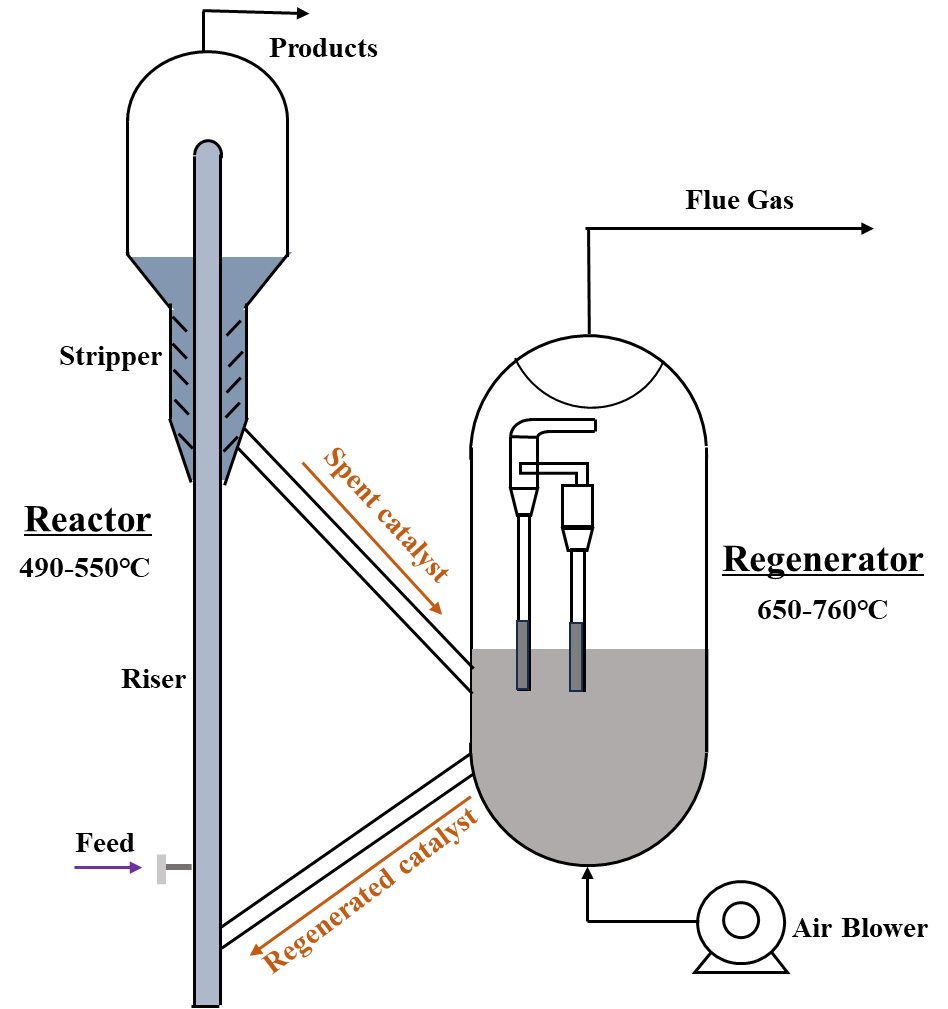


**Figure S1.** Schematic diagram of a typical industrial fluid catalytic cracking (FCC) unit.

1. **Monitoring of Flue Gas Composition Originating from Industrial Fluid Catalytic Cracking Units**

Three typical industrial fluid catalytic cracking (FCC) units (Table S1) with different regeneration processes were selected for the stack test described in this study. In industrial FCC units, the flue gas pollutants from the regenerator are purified by the tail treatment devices and then discharged into the air through the stack. In the middle of the stack, a monitoring window has been reserved for the manual monitoring of the flue gas, where the monitoring equipment is set.

Multiple monitoring methods were utilized for the sampling and measurement of 172 kinds of pollutants and three kinds of greenhouse gases. A Gasmet FT-IR DX-4000 analyzer was utilized for the field monitoring of CO_2_, CO, NO_2_, NO, N_2_O, SO_2_, NH_3_, HCN, CH_4_, benzene (C_6_H_6_), toluene (C_7_H_8_), styrene (C_8_H_8_), and ethylbenzene (C_8_H_10_). After nitrogen flushing and zero calibration, the flue gas is pumped into the equipment at a flow rate of 1 L/min. The FT-IR spectra of the flue gas were collected per 7 s, which were compared with the standard spectra of the database to provide the emission concentrations of pollutants. The detection limit was 0.01 ppm. A Laoying 3012H flue dust analyzer was used to collect filterable particulate matter (FPM) and condensable particulate matter (CPM) samples by filtration and condensation. These samples are weighed to calculate emission concentrations of FPM and CPM. Then, the filters were dissolved by aqua regia and analyzed by inductively coupled plasma-mass spectrometry (ICP-MS, NexION 2000, USA) to calculate the emission concentrations of metal hazardous air pollutants (HAPs), including As, Ba, Be, Cd, Cr (6^+^), Cr (total), Co, Cu, Mn, Mo, Ni, Sb, Se, V, and Zn. The injection flow rate was 1.0 mL/min, and the optical resolution was 0.009 nm. High-purity argon (99.99%) was used as carrier gas with a flow rate of 0.5 mL/min. The detection limit of metal HAPs was 0.01 mg/L for ICP-MS. Besides, other pollutants were also collected by the Teflon gas bags and glass adsorption tubes with activated carbon (Carbopack B) at the sampling sites and brought back to the laboratory for the latter analysis. Before sampling, the adsorption tubes were aged at 350℃ for 15 min under a 40 mL/min nitrogen flow. After sampling, the samples in the gas bags were injected into the analyzer while the samples in the adsorption tubes were thermally desorbed at 270℃ for 3 min under a 30 mL/min nitrogen flow into the analyzer. Volatile organic compounds (VOCs) were characterized by gas chromatography-mass spectrometry (GC-MS, 890A-5975C, Agilent Company, USA). High-purity nitrogen (99.999 %) was used as carrier gas. The inlet temperature of the GC was 200°C, and the column flow was 1.5 mL/min. The electron impact source was used for MS, and the ionization energy was 70 eV. The ion source temperature was 230°C, and the scan range was 35–270 amu. The detection limit was 0.001 mg/m^3^ for GC-MS. Dioxins, furans, and polychlorinated biphenyls (PCBs) were detected by isotope ratio-mass spectrometers (IR-MS, 253 Ultra, Thermo Fisher Scientific, USA). The inlet temperature was 200°C, and the carrier gas flow rate was 1.0 mL/min (99.999% helium). The ion of two monitoring peaks of pollutants was monitored by the selective ion monitoring (SIM) method, and the resolution of the ion peaks was more than 10000. The detection limit was 1 pg/m^3^ for IR-MS.

The field monitoring should be conducted under the normal operation of the units. After the instruments were connected, the airtightness check was conducted until the leakage was no more than 0.6 L/min. According to the isokinetic sampling method, the inlet gas speed of the sampling equipment was adjusted to match the flue gas flow rate. The parameters of the flue gas were recorded, including temperature, humidity, pressure, and gas composition. Then, the monitoring and sampling of pollutants began according to the relevant standards. On-site monitoring of some pollutants and greenhouse gases by FT-IR spectroscopy was repeated twice, 3 h each. The sampling of the other pollutants was repeated three times.

**Table S1.** Basic information of the three typical fluid catalytic cracking (FCC) units, labeled as U1, U2, and U3, located in China.

| Unit type | Purification device | Mass flow of feedstock oil (t/h) | Sulfur content in feedstock (g/kg) | Nitrogen content in feedstock (g/kg) | Volume flow of flue gas (m^3^/h) |
| --- | --- | --- | --- | --- | --- |
| U1 | WFGD | 177 | 2.40 | 2.55 | 184490 |
| U2 | SCR+WFGD | 109 | 2.87 | 2.87 | 177749 |
| U3 | SCR+WFGD | 142 | 2.61 | 3.00 | 276355 |

1. **Regulations and Standards for Sampling, Monitoring, and Analysis of the Fluid Catalytic Cracking Pollutants in Stack Tests**

GB/T 16157-1996. The determination of particulates and sampling methods of gaseous pollutants from exhaust gas of stationary source.

HJ 77.2-2008. Ambient air and waste gas Determination of polychlorinated dibenzo-p-dioxins (PCDDs) and polychlorinated dibenzofurans (PCDFs) Isotope dilution HRGC-HRMS.

HJ/T 397-2007. Technical specifications for emission monitoring of stationary source.

HJ 647-2013. Ambient air and stationary source emissions - Determination of gas and particle-phase polycyclic aromatic hydrocarbons - High performance liquid chromatography

HJ 734-2014. Stationary source emission -Determination of volatile organic compounds-Sorbent adsorption and thermal desorption gas chromatography mass spectrometry method.

HJ 777-2015. Ambient air and waste gas from stationary sources emission -Determination of metal elements in ambient particle matter-Inductively coupled plasma optical emission spectrometry.

HJ 1153-2020. Stationary source emission - Determination of aldehyde and ketone compounds - Solution absorption - High performance liquid chromatography.

Method 202. Dry impinger method for determining condensable particulate emissions from stationary sources.

Method 1668C. Chlorinated biphenyl congeners in Water, soil, sediment, biosolids, and tissue by HRGC/HRMS.

1. **Characterization of the Spent Catalyst Materials Originating from the Industrial Fluid Catalytic Cracking Units**

The spent FCC materials (labeled as Cat_1_, Cat_2_, and Cat_3_) were directly collected from the three industrial FCC units under study and pretreated before the experiments in the laboratory. After the catalyst was separated from the product mixture, 500 g of spent catalyst materials were collected in the circulating inclined pipe from the reactor to the regenerator. The catalyst materials were dried at 80°C for 24 h and stored in a desiccator. Particularly, the particles of Cat_1_ clumped together, and the sample was sieved (125-250 μm) to obtain a fine catalyst powder.

The elemental content (i.e., C, H, N, and S) of coke deposits on the catalyst materials was determined by an Elementar Vario El Cube elemental analyzer. 2 mg of the spent catalyst material was placed in the combustion chamber and then heated to 1200°C in an O_2_ atmosphere. The gas products were separated through an adsorption-desorption column and detected by a thermal conductivity detector. According to the concentration of gas products, the elemental content was calculated.

X-ray photoelectron spectroscopy (XPS) characterization was performed on a Thermo Scientific K-Alpha X-ray photoelectron spectrometer, using Al Kα non-monochromatic radiation (i.e., 1486.6 eV). The binding energy (BE) spectra of the C1s, N1s, and S2p regions were peak-fitted with the XPSPEAK software using a Shirley background correction and a Gaussian-Lorentzian (80/20) function. The BE of C1s at 284.8 eV was used as an internal standard to calibrate the BEs of other chemical elements.

Gas chromatography-mass spectrometry (GC-MS) analysis was carried out on an Agilent 890A-5975C GC-MS instrument. Before performing the GC-MS measurements, the spent catalyst materials were dissolved by HF acid to release trapped coke deposits and then extracted by the Soxhlet extraction method. 30 mL HF acid (Sinopharm Chemical Reagent, 40 wt.%) and 10 mL HCl acid (Sinopharm Chemical Reagent, 36 wt.%) were poured into a Teflon beaker. 10 g of the spent catalyst material was slowly added to the acid solution and left at room temperature for 24 h. The saturated Na_2_CO_3_ (Aladdin Chemical Reagent, 99.5%) solution was dropped into the above solution until the pH value was 8-9. Then, the sample was filtered, washed, and dried. The sample was wrapped in filter paper and placed in a Soxhlet extractor. 400 mL of CH_2_Cl_2_ (Aladdin Chemical Reagent, 99.5%) was added to the flask at the bottom and heated at 40°C for 24 h with a reflux speed of 10-12 times/h. Finally, the extraction solution was transferred to a rotary evaporator and concentrated to 5 mL for GC-MS analysis.

Thermogravimetric analysis (TGA) experiments were carried out on a PerkinElmer TGA 8000 TGA instrument. Approximately 1.5 mg of the spent catalyst material was placed in the furnace and heated from 30℃ to 700℃ at a heating rate of 10℃/min. The feed gas included 0, 10, and 20 vol.% O_2_/N_2_. The weight loss was recorded with an accuracy of 0.1 µg. The differential thermogravimetric (DTG) curve is obtained by differentiating the TG curve.

Confocal fluorescence microscopy (CFM) images of the spent and regenerated catalyst materials were acquired using a Nikon Eclipse 90i confocal microscope with a 100 × 0.73 NA dry objective. The excitation light was provided by focusing four different lasers (i.e., 408 nm, 488 nm, 561 nm, and 642 nm) simultaneously on the catalyst particle located in an open measurement cell (Linkam Instruments, FTIR 600). The emitted light from the sample was collected by a Nikon A1 detector equipped with photomultiplier tubes in the range of 450-700 nm, with a resolution of 6 nm. The two-dimensional microscopy slices at different focal depths were used to reconstruct the three-dimensional spatial distribution of the fluorescent species (which are part of the coke deposits) in the FCC particles.

1. **Regeneration of the Spent Catalyst Materials**

The regeneration experiments were performed at different temperatures ranging from 30°C to 700°C under different gas atmospheres (i.e., 0, 10, and 20 vol.% O_2_/N_2_). *In situ* Raman and *operando* FT-IR spectroscopy were used to analyze the evolution of coke species, coupled with online FT-IR spectroscopy to monitor the emission of the gas pollutants, as shown in Figure S2.


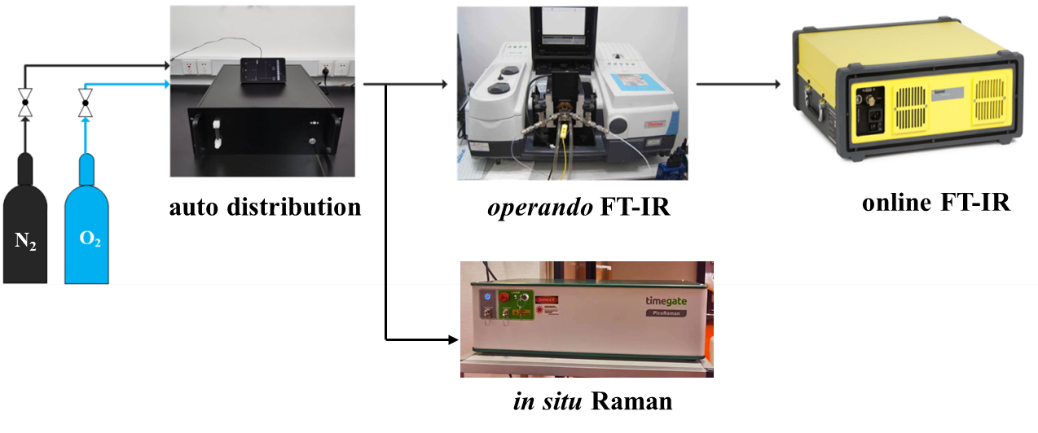


**Figure S2.** Schematic diagram of an *in situ* and *operando* spectroscopic system for the regeneration of spent fluid catalytic cracking (FCC) materials.

*In situ* Raman spectroscopy measurements were performed on an upright Olympus microscope (Olympus BX41M) mounted with a Raman Microprobe (Timegate Instruments). The Microprobe was fiber-coupled to a Ventus 532 nm continuous wave laser with a mpc6000 power supply unit (Novanta Photonics) and an AvaRaman-532 TEC (Avantes) spectrometer equipped with a 25 μm slit, and a back-thinned thermoelectrically cooled CCD detector with 1024 × 58 pixels. A 50 × 0.5 NA Olympus objective with a working distance of 10.6 mm was used to deliver 1.3 mW to the sample, measured with an Ophir Orion PD power meter and a PD300 photodiode head. The laser illuminated a spherical area of 41 μm in diameter, suitable to probe a significant fraction of single FCC catalyst particles. Overview reflection images of the three samples under study were recorded with a CS165CU/M 1.6 MP Color CMOS Camera (Zelux) that was integrated in the Microprobe, using a 10 × 0.3 NA Olympus objective with a working distance of 10 mm.

For each regeneration experiment probed with *in situ* Raman microscopy, 10 mg of the coked catalyst powder was loaded in a TS1200 Linkam stage (Linkam Scientific) and placed underneath the microscope. The spent catalyst material was heated up from 20 to 700°C at a rate of 20°C/min under the flow of a mixture of x% O_2_ and 100-x% N_2_ (x = 0, 10, or 20%) (10 mL/min). Before the start of the regeneration process, we selected an FCC particle that exhibited distinct D- and G-bands. During catalyst regeneration, a Raman spectrum of the selected particle was recorded every 12 s (250 ms integration time, and averaged 48 times). The spectral recording was preceded by a background acquisition in the absence of the laser source. The samples were regenerated using three measurement conditions. Each experiment was repeated three times, resulting in a total of 27 experiments. The Raman spectra are presented after background subtraction and a correction for the pixel response of the detector and the transmission of the microscope optics.

*Operando* FT-IR spectroscopy was developed with online FT-IR spectroscopy measurement of gas products during the catalyst regeneration experiments. *Operando* FT-IR spectroscopy experiments were carried out on a Thermo Nicolet-6700 FT-IR spectrometer with an MCT-B detector cooled by liquid N_2_. Approximately 100 mg of spent catalyst powder was placed in the sample cell. Under the gas flow of 10 mL/min, the regeneration temperature rose from 30℃ to 700℃ at a heating rate of 10℃/min. The spectra were recorded from the range of 4000-700 cm^−1^ with a spectral resolution of 4 cm^−1^ and 64 scans per sampling. In addition, the gas products in regeneration experiments were measured by online FT-IR spectroscopy. The online FT-IR spectra of the outlet gas pollutants of industrially used FCC units were recorded with a Gasmet Dx-4000 FTIR gas analyzer. For each spectrum, 64 scans were run from the range of 4000-900 cm^−1^ with a resolution of 8 cm^−1^. The sample cell was heated up to 180℃ to prevent gas condensation. The concentrations of gas pollutants were provided by the Calcmet software.

**Table S2. The detection range and method detection limits of gas pollutants.**

| **CAS Number** | **Pollutants** | **Unit** | **Range** | **Detection limit in N_2_** |
| --- | --- | --- | --- | --- |
| 14485-07-5 | Carbon dioxide (CO_2_) | vol.% | 0-30 | NP |
| 630-08-0 | Carbon monoxide (CO) | ppm | 0-30000 | 0.222 |
| 7446-09-5 | Sulfur dioxide (SO_2_) | ppm | 0-3000 | 0.026 |
| 10102-44-0 | Nitrogen dioxide (NO_2_) | ppm | 0-500 | 0.329 |
| 10102-43-9 | Nitric oxide (NO) | ppm | 0-2000 | 0.256 |
| 7664-41-7 | Ammonia (NH_3_) | ppm | 0-1000 | 0.156 |
| 74-90-8 | Hydrogen cyanide (HCN) | ppm | 0-500 | NP |
| 7439-92-1 | Methane (CH_4_) | ppm | 0-10000 | 0.105 |
| 115-07-1 | Propylene (C_3_H_6_) | ppm | 0-200 | 0.220 |
| 107-01-7 | Butene (C_4_H_8_) | ppm | 0-200 | 0.093 |
| 108-88-3 | Toluene (C_7_H_8_) | ppm | 0-100 | NP |
| 100-41-4 | Ethylbenzene (C_8_H_10_) | ppm | 0-100 | NP |

NP: Not provided by the manufacturer.

1. **Theoretical Calculations**

The calculations were based on Density Functional Theory (DFT) and performed by using the Gaussian 09 program^[1]^. The geometrical structures were optimized at the B3LYP/6-31G* level, and the normal modes of vibrations of these structures were computed to verify them as energy minima. Single-point energies for all the molecules were calculated at the B2PLYP/def2-TZVP level on the basis of geometry optimizations.

2. Supplementary Results and Discussion

S1. Emission Concentrations of Pollutants and Greenhouse Gases in the Flue Gases of Industrial Fluid Catalytic Cracking Units

**Table S3.** Monitoring results of gas pollutants (mg/m^3^) in the three different industrial fluid catalytic cracking (FCC) units under study.

| **CAS Number or Pollutant Code** | **Pollutants** | **U1** | **U2** | **U3** |
| --- | --- | --- | --- | --- |
| PM25-FIL | FPM ≤ 2.5 µm | 1.03E+02 | 3.10E+01 | 1.01E+02 |
| PM-CON | Condensable PM | 1.21E+01 | 8.61 | 2.03E+01 |
| PM25-PRI | PM ≤ 2.5 µm | 1.15E+02 | 3.97E+01 | 1.21E+02 |
| 7446-09-5 | Sulfur dioxide | 1.42E+02 | 2.81E+01 | 1.19E+01 |
| 10102-43-9 | Nitric oxide | 3.24E+01 | 5.48E+01 | 1.07E+01 |
| 10102-44-0 | Nitrogen dioxide | 1.59 | 3.1E-01 | 2.06E+01 |
| 630-08-0 | Carbon monoxide | 2.99E+04 | 2.74E+03 | 9.18E+02 |
| 7439-92-1 | Lead | 3.42E-03 | 7.47E-03 | 8.37E-03 |
| 74-85-1 | Ethylene | ND | ND | ND |
| 74-86-2 | Acetylene | ND | ND | ND |
| 74-98-6 | Propane | ND | ND | 2.25 |
| 115-07-1 | Propylene | 1.28E+01 | 1.40E-01 | 3.00E-02 |
| 463-49-0 | Propylene | ND | ND | ND |
| 106-97-8 | n-Butane | ND | ND | ND |
| 75-28-5 | Isobutane | ND | ND | ND |
| 106-98-9 | 1-Butene | ND | ND | ND |
| 107-01-7 | 2-Butene | 9.10E-01 | 3.30E-01 | 2.00E-02 |
| 115-11-7 | Isobutene | ND | 8.30E-01 | 1.39 |
| 590-19-2 | 1,2-Butadiene | ND | ND | ND |
| 109-66-0 | n-pentane | ND | ND | ND |
| 78-78-4 | 2-Methylbutane | ND | ND | ND |
| 287-92-3 | Cyclopentane | ND | ND | ND |
| 591-95-7 | 1,2-Pentadiene | ND | ND | ND |
| 1574-41-0 | 1-cis-3-Pentadiene | ND | ND | ND |
| 2004-70-8 | 1-trans-3-Pentadiene | ND | ND | ND |
| 591-93-5 | 1,4-Pentadiene | ND | ND | ND |
| 591-96-8 | 2,3-Pentadiene | ND | ND | ND |
| 598-25-4 | 3-Methyl-1,2-butadiene | ND | ND | ND |
| 78-79-5 | 2-Methyl-1,3-butadiene | ND | ND | ND |
| 542-92-7 | Cyclopentadiene | ND | ND | ND |
| 110-82-7 | Cyclohexane | ND | ND | ND |
| 108-87-2 | Methylcyclohexane | ND | ND | ND |
| 142-82-5 | Heptane (and isomers) | ND | ND | ND |
| 111-65-9 | Octane (and isomers) | ND | ND | ND |
| 78-93-3 | Methyl ethyl ketone | 1.39E-02 | 1.29E-02 | 2.95E-02 |
| 25551-13-7 | Trimethylbenzene(s) | ND | ND | ND |
| 75-07-0 | Acetaldehyde | 2.00E-01 | 2.30E-01 | 2.00E-01 |
| 107-02-8 | Acrolein | ND | ND | ND |
| 62-53-3 | Analine | ND | ND | ND |
| 71-43-2 | Benzene | 1.35E-02 | 8.30E-03 | 2.89E-02 |
| 74-83-9 | Bromomethane | ND | ND | ND |
| 106-99-0 | 1,3-Butadiene | ND | ND | ND |
| 75-00-3 | Chloroethane | ND | ND | ND |
| 67-66-3 | Chloroform | 1.20E-03 | ND | 1.08E-01 |
| 74-87-3 | Chloromethane | 1.37E-01 | 2.24E-01 | 2.43E-01 |
| 98-82-8 | Cumene | 1.78E-02 | ND | 6.20E-03 |
| 106-93-4 | 1,2-Dibromoethane | 1.30E-03 | ND | 3.20E-03 |
| 106-46-7 | 1,4-Dichlorobenzene | 8.90E-03 | 7.33E-02 | 2.97E-02 |
| 75-34-3 | 1,1-Dichloroethane | ND | ND | ND |
| 107-06-2 | 1,2-Dichloroethane | 3.60E-03 | 1.50E-03 | 8.30E-03 |
| 75-35-4 | 1,1-Dichloroethylene | ND | ND | ND |
| 78-87-5 | 1,2-Dichloropropane | 7.40E-03 | ND | 5.40E-03 |
| 542-75-6 | 1,3-Dichloropropene | ND | ND | ND |
| 111-42-2 | Diethanolamine | ND | ND | ND |
| 100-41-4 | Ethylbenzene | 3.55E+02 | 1.00E-01 | 7.00E-02 |
| 50-00-0 | Formaldehyde | 2.90E-01 | 4.00E-01 | 5.50E-01 |
| 67-72-1 | Hexachloroethane | ND | ND | ND |
| 110-54-3 | n-Hexane | ND | ND | 1.30E-03 |
| 67-56-1 | Methanol | ND | ND | 8.30 |
| 108-10-1 | Methyl isobutyl ketone | 1.60E-03 | 1.53E-01 | 8.80E-03 |
| 1634-04-4 | Methyl tert-butyl ether | ND | ND | ND |
| 100-42-5 | Styrene | 5.69E+01 | 3.26E+01 | 1.30E+01 |
| 79-34-5 | 1,1,2,2-  Tetrachloroethane | 4.20E-03 | 3.55E-02 | 2.10E-03 |
| 127-18-4 | Tetrachloroethylene | 1.70E-03 | 1.14E-02 | 4.77E-02 |
| 108-88-3 | Toluene | 4.96E+01 | 7.00E-02 | 1.10E-01 |
| 79-00-5 | 1,1,2-Trichloroethane | 6.40E-03 | ND | 2.80E-03 |
| 79-01-6 | Trichloroethylene | 1.80E-03 | ND | 6.10E-03 |
| 121-44-8 | Triethylamine | ND | ND | ND |
| 540-84-1 | 2,2,4-Trimethylpentane | ND | ND | ND |
| 593-60-2 | Vinyl bromide | ND | ND | ND |
| 95-47-6 | o-Xylene | 2.56E-02 | 2.01E-02 | 8.00E-03 |
| 108-38-3 | m-Xylene | 1.46E-02 | 1.23E-02 | 1.43E-02 |
| 106-42-3 | p-Xylene |  |  |  |
| 1330-20-7 | Xylenes (total) | 2.56E-02 | 2.01E-02 | 2.20E-02 |
| 83-32-9 | Acenaphthene | 4.99E-01 | 5.33E-01 | 4.67E-01 |
| 208-96-8 | Acenaphthylene | ND | ND | ND |
| 120-12-7 | Anthracene | 2.40E-04 | 1.00E-05 | 1.40E-04 |
| 56-55-3 | Benzo(a)anthracene | ND | ND | ND |
| 50-32-8 | Benzo(a)pyrene | ND | ND | ND |
| 205-99-2 | Benzo(b)fluoranthene | ND | ND | ND |
| 192-97-2 | Benzo(e)pyrene | ND | ND | ND |
| 191-24-2 | Benzo(g,h,i)perylene | ND | ND | ND |
| 207-08-9 | Benzo(k)fluoranthene | ND | ND | ND |
| 92-52-4 | Biphenyl | ND | ND | ND |
| 117-81-7 | Bis(2-ethyl hexyl)  phthalate | 7.52E-02 | 8.07E-02 | 7.75E-02 |
| 91-58-7 | 2-Chloronaphthalene | ND | ND | ND |
| 108-39-4 | m-Cresol | ND | ND | ND |
| 95-48-7 | o-Cresol | ND | ND | ND |
| 106-44-5 | p-Cresol | ND | ND | ND |
| 1319-77-3 | Cresols (total) | ND | ND | ND |
| 53-70-3 | Dibenz(a,h)anthracene | ND | ND | ND |
| 84-74-2 | di-n-Butyl phthalate | 7.15E-02 | 9.06E-02 | 6.87E-02 |
| 84-66-2 | Diethyl-phthalate | 1.85E-01 | 4.85E-01 | 3.05E-01 |
| 57-97-6 | 7,12-Dimethylbenz(a)  anthracene | ND | ND | ND |
| 206-44-0 | Fluoranthene | 4.70E-04 | 7.40E-04 | 1.08E-03 |
| 86-73-7 | Fluorene | ND | ND | ND |
| 193-39-5 | Indeno(1,2,3-cd)  pyrene | ND | ND | ND |
| 56-49-5 | 3-Methylchloranthrene | ND | ND | ND |
| 91-57-6 | 2-Methylnaphthalene | 1.50 | 1.64 | 1.41 |
| 218-01-9 | Chrysene | 1.00E-05 | 3.00E-05 | 1.00E-04 |
| 91-20-3 | Naphthalene | 9.44E-02 | 1.38E-01 | 9.23E-02 |
| 198-55-0 | Perylene | ND | ND | ND |
| 85-01-8 | Phenanthrene | 4.83E-04 | 8.97E-04 | 4.30E-04 |
| 108-95-2 | Phenol | ND | ND | ND |
| 129-00-0 | Pyrene | ND | 9.00E-05 | 3.00E-05 |
| 1746-01-6 | 2,3,7,8-T_4_CDD | 5.00E-10 | 6.00E-10 | 5.33E-10 |
| 40321-76-4 | 1,2,3,7,8-P_5_CDD | 2.20E-09 | 3.25E-09 | 8.95E-09 |
| 39227-28-6 | 1,2,3,4,7,8-H_6_CDD | 2.55E-09 | 2.77E-09 | 3.80E-09 |
| 57653-85-7 | 1,2,3,6,7,8-H_6_CDD | 2.53E-09 | 3.03E-09 | 2.93E-09 |
| 19408-74-3 | 1,2,3,7,8,9-H_6_CDD | 2.53E-09 | 1.87E-09 | 4.10E-09 |
| 35822-46-9 | 1,2,3,4,6,7,8-H_7_CDD | 1.72E-08 | 9.37E-09 | 1.38E-08 |
| 3268-87-9 | O_8_CDD | 4.00E-08 | 1.80E-08 | 2.80E-08 |
| 132-64-9 | Dibenzofuran | 4.39E-05 | 2.80 E-05 | 2.30E-05 |
| 51207-31-9 | 2,3,7,8-T_4_CDF | 4.65E-09 | 5.33E-09 | 2.51E-07 |
| 57117-41-6 | 1,2,3,7,8-P_5_CDF | 7.05E-09 | 7.00E-09 | 4.80E-09 |
| 57117-31-4 | 2,3,4,7,8-P_5_CDF | 1.03E-08 | 1.96E-08 | 1.14E-08 |
| 70648-26-9 | 1,2,3,4,7,8-H_6_CDF | 1.30E-08 | 8.67E-09 | 3.77E-09 |
| 57117-44-9 | 1,2,3,6,7,8-H_6_CDF | 1.22E-08 | 1.02E-08 | 1.85E-09 |
| 60851-34-5 | 2,3,4,6,7,8-H_6_CDF | 1.38E-08 | 1.61E-08 | 4.13E-09 |
| 72918-21-9 | 1,2,3,7,8,9-H_6_CDF | 3.83E-09 | 4.97E-09 | 1.80E-09 |
| 67562-39-4 | 1,2,3,4,6,7,8-H7CDF | 5.31E-08 | 2.10E-08 | 9.77E-08 |
| 55673-89-7 | 1,2,3,4,7,8,9-H_7_CDF | 5.90E-09 | 3.40E-09 | 1.20E-09 |
| 39001-02-0 | O_8_CDF | 2.71E-08 | 7.03E-09 | 4.27E-09 |
| 70362-50-4 | PCB81 | 2.10E-09 | 1.44E-08 | 2.23E-08 |
| 32598-13-3 | PCB77 | 1.95E-08 | 4.48E-08 | 1.66E-06 |
| 57465-28-8 | PCB126 | 5.18E-09 | 3.83E-08 | 7.32E-08 |
| 32774-16-6 | PCB169 | 1.30E-09 | 1.72E-08 | 2.69E-08 |
| 65510-44-3 | PCB123 | 1.28E-09 | 7.33 E-10 | 1.13E-08 |
| 31508-00-6 | PCB118 | 2.29E-08 | 1.54E-08 | 6.28E-07 |
| 32598-14-4 | PCB105 | 7.10E-09 | 1.66E-08 | 6.50E-08 |
| 74472-37-0 | PCB114 | 1.38E-06 | 7.67 E-10 | 7.57E-09 |
| 52663-72-6 | PCB167 | 3.38E-09 | 2.07 E-09 | 1.32E-08 |
| 38380-08-4 | PCB156 | 5.78E-09 | 5.93E-09 | 8.77E-09 |
| 69782-90-7 | PCB157 | 1.85E-09 | 9.75E-09 | 5.20E-09 |
| 39635-31-9 | PCB189 | 4.37E-09 | 4.97E-09 | 1.00E-09 |
| 7012-37-5 | PCB28 | 3.84E-07 | 1.89E-07 | 3.15E-07 |
| 35693-99-3 | PCB52 | 9.90E-08 | 5.68E-08 | 1.07E-07 |
| 37680-73-2 | PCB101 | 4.01E-08 | 2.05E-08 | 8.83E-08 |
| 35065-28-2 | PCB138 | 3.41E-08 | 1.16E-08 | 9.30E-09 |
| 35065-27-1 | PCB153 | 5.06E-08 | 1.37E-08 | 2.66E-08 |
| 35065-29-3 | PCB180 | 3.05E-08 | 1.59E-08 | 9.53E-09 |
| PCB-3 | PCB16 - 39 | 1.03E-06 | 6.30E-07 | 1.13E-06 |
| PCN-4 | PCB40 - 81 | 1.62E-06 | 1.36E-06 | 1.63E-05 |
| PCB-5 | PCB82 - 127 | 2.12E-07 | 1.79E-07 | 2.51E-06 |
| PCB-6 | PCB128 - 169 | 2.66E-07 | 1.38E-07 | 5.66E-07 |
| PCB-7 | PCB170 - 193 | 1.54E-07 | 8.04E-08 | 9.16E-08 |
| PCB-8 | PCB194 - 205 | 1.10E-08 | 2.94E-08 | 1.77E-08 |
| PCB-9 | PCB206 - 208 | 4.63E-09 | 1.39E-09 | 6.87E-09 |
| 2051-24-3 | PCB209 | 8.68E-09 | 8.47E-09 | 3.10E-09 |
| 1336-36-3 | Polychlorinated biphenyl (total) | 3.33E-06 | 2.47E-06 | 2.07E-05 |
| 7440-36-0 | Antimony | 2.36E-03 | 1.53E-03 | 8.63E-04 |
| 7440-38-2 | Arsenic | 2.53E-04 | 2.8 E-04 | 3.30E-04 |
| 7440-41-7 | Beryllium | 3.46E-05 | 3.39E-05 | 2.15E-05 |
| 7440-43-9 | Cadmium | 1.22E-04 | 5.12E-02 | 1.06E-05 |
| 18540-29-9 | Chromium (hexavalent) | ND | ND | ND |
| 7440-47-3 | Chromium (total) | 2.06E-02 | 1.34E-03 | 5.17E-02 |
| 7440-48-4 | Cobalt | 1.15E-03 | 2.99E-04 | 3.33E-03 |
| 7439-96-5 | Manganese | 1.70E-02 | 3.10E-03 | 1.70E-02 |
| 7440-02-0 | Nickel | 7.10E-02 | 7.57E-02 | 1.43E-01 |
| 7782-49-2 | Selenium | 5.37E-03 | 2.41E-03 | 1.63E-03 |
| 7647-01-0 | Hydrogen chloride | 2.44 | 4.54 | 4.20 |
| 74-90-8 | Hydrogen cyanide | 1.09E+02 | 2.83 | 1.00E-02 |
| 7664-41-7 | Ammonia | 2.16E+02 | 3.41 | 1.75 |
| 7440-39-3 | Barium | 7.45E-03 | 7.62E-03 | 7.03E-03 |
| 7440-50-8 | Copper | 1.96E-03 | 2.88E-03 | 1.85E-03 |
| 7439-98-7 | Molybdenum | 2.14E-03 | 1.77E-03 | 4.07E-03 |
| 7440-62-2 | Vanadium | 3.70E-02 | 2.05E-02 | 9.87E-03 |
| 7440-66-6 | Zinc | 2.01E-02 | 2.18E-03 | 3.17E-03 |

**Table S4**. Monitoring results of three greenhouse gases CO_2_, CH_4_, and N_2_O (mg/m^3^) in the three industrial fluid catalytic cracking (FCC) units under study.

| **CAS Number or Pollutant Code** | **Greenhouse gases** | **U1** | **U2** | **U3** |
| --- | --- | --- | --- | --- |
| 14485-07-5 | CO_2_ | 1.32E+05 | 1.42E+05 | 1.21E+05 |
| 7439-92-1 | CH_4_ | 5.85E+02 | 1.96 | 0 |
| 10024-97-2 | N_2_O | 1.36E+06 | 9.98 | 2.61E+01 |

S2. Coke Deposits on the Spent Catalyst Materials

**Table S5**. Main hydrocarbons, as observed in the gas chromatography-mass spectrometry (GC-MS) data for the three different fluid catalytic cracking (FCC) materials under study.

| **Cat_1_** | |  |  | **Cat_2_** | |  |  | **Cat_3_** | |  |
| --- | --- | --- | --- | --- | --- | --- | --- | --- | --- | --- |
| **Structure** | **Ratio/%** |  |  | **Structure** | **Ratio/%** |  |  | **Structure** | **Ratio/%** |  |
| **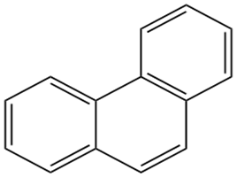**  **C_14_H_10_** | **24.53** |  |  | **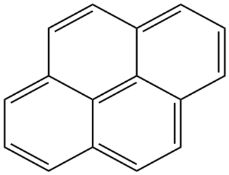**  **C_16_H_10_** | **18.65** |  |  | **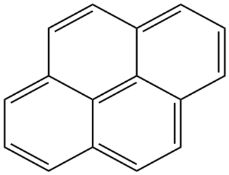**  **C_16_H_10_** | **17.51** |  |
| **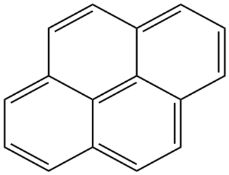**  **C_16_H_10_** | **21.28** |  |  | **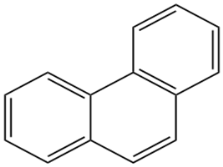**  **C_14_H_10_** | **9.54** |  |  | **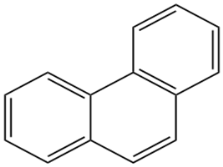**  **C_14_H_10_** | **10.93** |  |
| **Nonadecene**  **C_19_H_38_** | **6.5** |  |  | **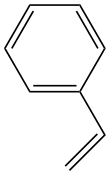**  **C_8_H_8_** | **9.23** |  |  | **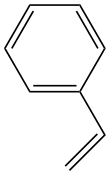**  **C_8_H_8_** | **10.61** |  |
| **Octane**  **C_8_H_16_** | **4.34** |  |  | **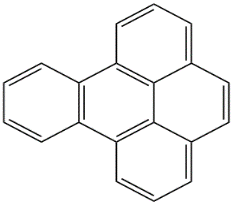**  **C_20_H_12_** | **7.43** |  |  | **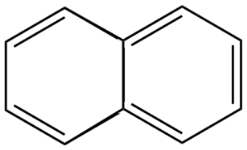**  **C_10_H_8_** | **8.02** |  |
| **Tridecane**  **C_13_H_28_** | **2.06** |  |  | **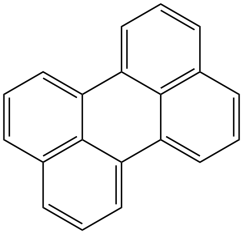**  **C_20_H_12_** | **4.27** |  |  | **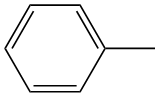** | **5.62** |  |
| **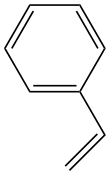**  **C_8_H_8_** | **1.96** |  |  | **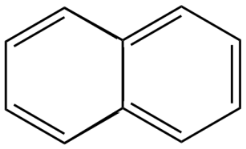**  **C_10_H_8_** | **4.15** |  |  | **Tridecane**  **C_13_H_28_** | **2.81** |  |
| **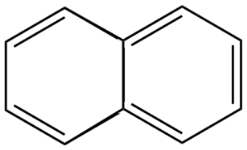**  **C_10_H_8_** | **1.89** |  |  | **1-Cetene**  **C_16_H_32_** | **3.82** |  |  | **Hexadecane**  **C_16_H_34_** | **2.53** |  |

**Table S6.** Main oxygen-containing compounds, as observed in the gas chromatography-mass spectrometry (GC-MS) data for the three different fluid catalytic cracking (FCC) materials under study.

| **Cat_1_** | |  |  | **Cat_2_** | |  |  | **Cat_3_** | | |  |
| --- | --- | --- | --- | --- | --- | --- | --- | --- | --- | --- | --- |
| **Structure** | **Ratio/%** |  |  | **Structure** | **Ratio/%** |  |  | **Structure** | **Ratio/%** |  |  |
| **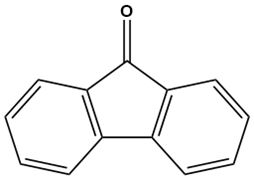**  **C_13_H_8_O** | **3.88** |  |  | **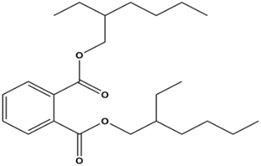**  **C_24_H_38_O_4_** | **2.05** |  |  | **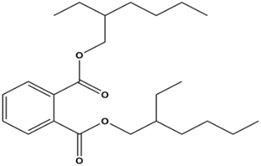**  **C_24_H_38_O_4_** | **2.06** |  |  |
| **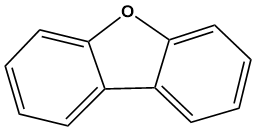**  **C_12_H_8_O** | **3.61** |  |  | **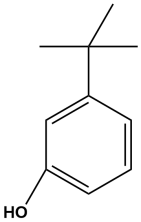**  **C_10_H_14_O** | **1.19** |  |  | **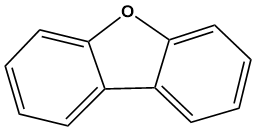**  **C_12_H_8_O** | **1.06** |  |  |
| **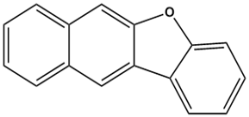**  **C_15_H_8_O** | **1.39** |  |  | **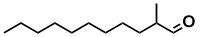**  **C_12_H_24_O** | **0.99** |  |  | **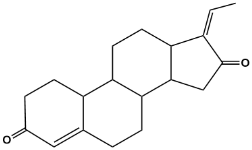**  **C_21_H_28_O_2_** | **0.92** |  |  |
| **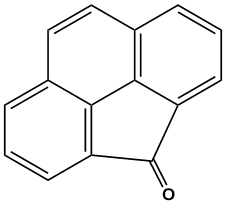**  **C_15_H_8_O** | **1.24** |  |  | **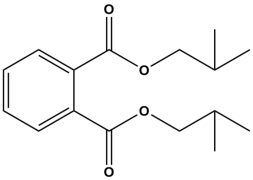**  **C_16_H_22_O_4_** | **0.89** |  |  | **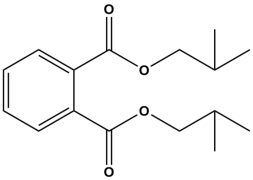**  **C_16_H_22_O_4_** | **0.84** |  |  |
| **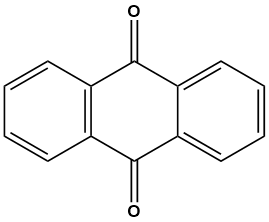**  **C_14_H_8_O_2_** | **1.08** |  |  |  |  |  |  | **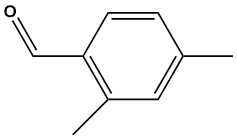**  **C_9_H_10_O** | **0.58** |  |  |

**Table S7.** Main nitrogen-containing compounds, as observed in the gas chromatography-mass spectrometry (GC-MS) data for the three different fluid catalytic cracking (FCC) materials under study.

| **Cat_1_** | | | **Cat_2_** | | | | **Cat_3_** | | |
| --- | --- | --- | --- | --- | --- | --- | --- | --- | --- |
| **Structure** | **Ratio/%** |  |  | **Structure** | **Ratio/%** |  |  | **Structure** | **Ratio/%** |
| **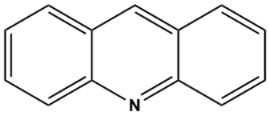**  **C_13_H_9_N** | **1.7** |  |  | **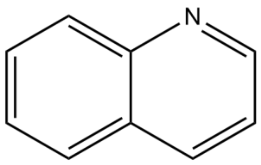**  **C_9_H_7_N** | **7.3** |  |  | **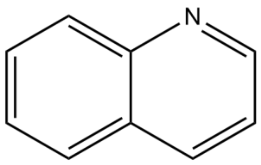**  **C_9_H_7_N** | **3.67** |
| **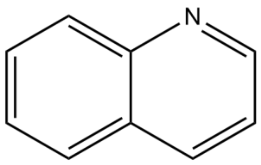**  **C_9_H_7_N** | **0.71** |  |  | **9-Octadecenoamide**  **C_18_H_32_NO** | **4.27** |  |  | **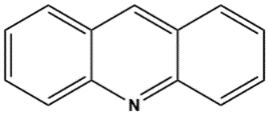**  **C_13_H_9_N** | **2.18** |
| **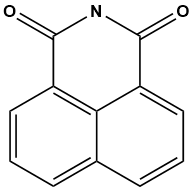**  **C_12_H_7_NO_2_** | **0.70** |  |  | **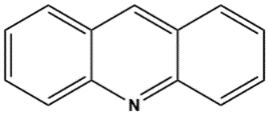**  **C_13_H_9_N** | **2.18** |  |  | **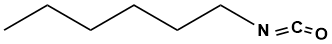**  **C_7_H_13_NO** | **1.7** |
| **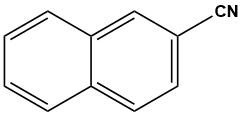**  **C_11_H_7_N** | **0.32** |  |  | **13-Docosenoamide**  **C_22_H_43_NO** | **1.53** |  |  | **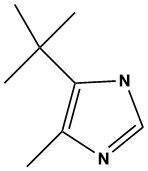**  **C_8_H_14_N_2_** | **0.94** |
| **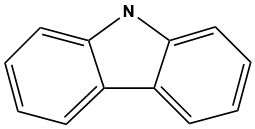**  **C_12_H_9_N** | **0.30** |  |  | **Hexadecanamide**  **C_16_H_33_NO** | **1.22** |  |  | **Hexadecanamide**  **C_16_H_33_NO** | **0.62** |
| **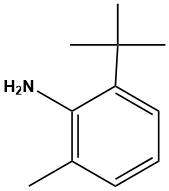**  **C_11_H_17_N** | **0.24** |  |  | **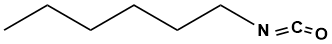**  **C_7_H_13_NO** | **1.19** |  |  | **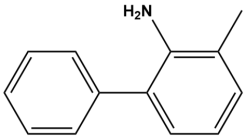**  **C_13_H_15_N** | **0.41** |

**Table S8.** Main sulfur-containing compounds, as observed in the gas chromatography-mass spectrometry (GC-MS) spectra for the three different fluid catalytic cracking (FCC) materials under study.

| **Cat_1_** | |  | **Cat_2_** | |  | **Cat_3_** | |  |
| --- | --- | --- | --- | --- | --- | --- | --- | --- |
| **Structure** | **Ratio/%** |  | **Structure** | **Ratio/%** |  | **Structure** | **Ratio/%** |  |
| **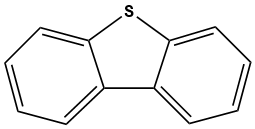**  **C_12_H_8_S** | **2.27** |  | **-** | **-** |  | **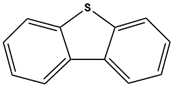C_12_H_8_S** | **1.78** |  |
| **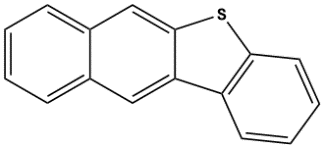**  **C_16_H_10_S** | **0.22** |  | **-** | **-** |  | **-** | **-** |  |


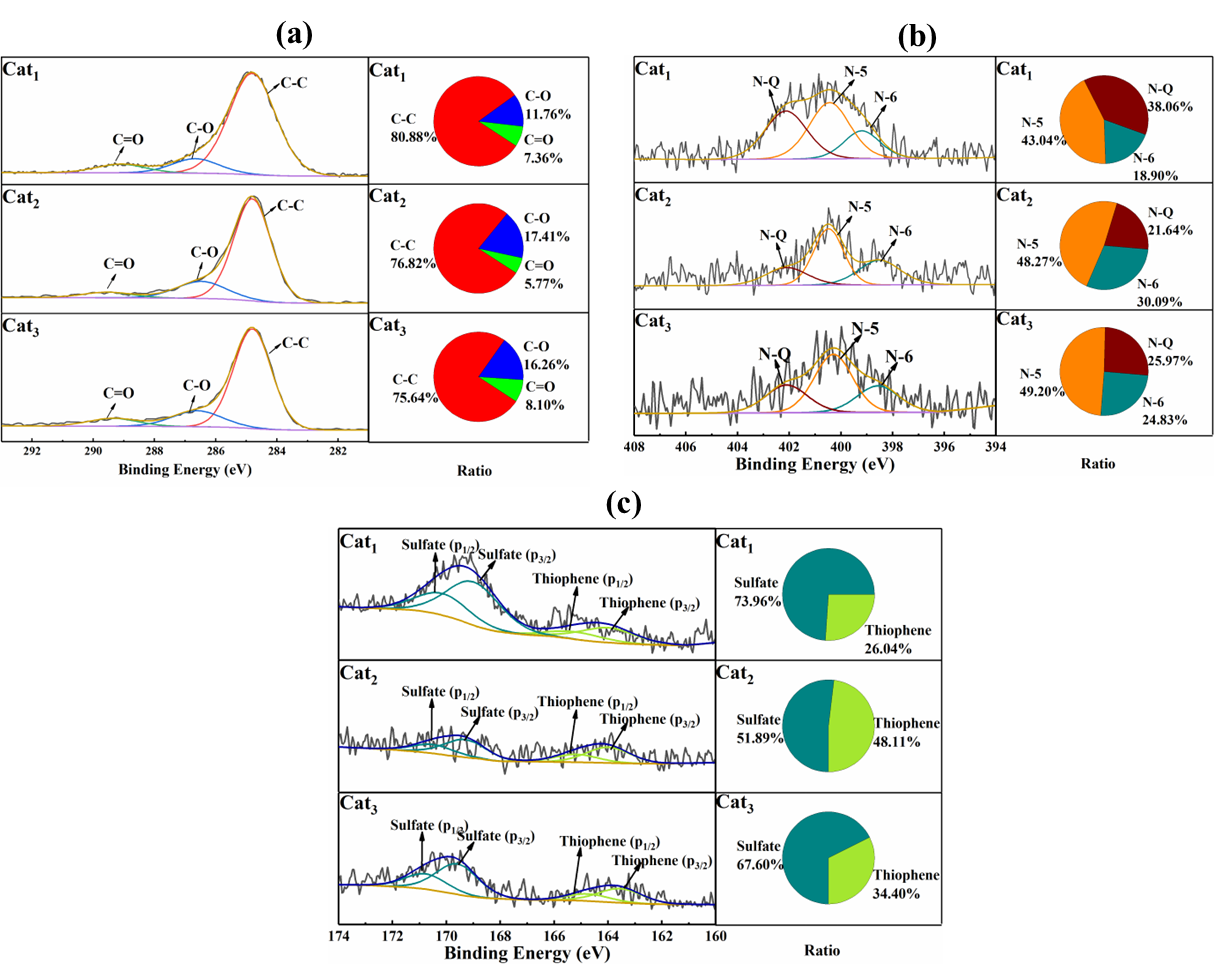


**Figure S3.** X-ray photoelectron spectroscopy (XPS) data of the three fluid catalytic cracking (FCC) spent materials under study. **(a)**, C1s. **(b)**, N1s. **(c)**, S2p spectral region.

For the nitrogen species in the coke deposits, the results originating from the GC-MS and XPS measurements are unfortunately inconsistent to a certain extent. Except for quinoline/acridine and carbazole, other nitrogen-containing compounds (i.e., imidazole, aniline, nitrile, isocyanate, and amide) detected by GC-MS, but are not seen in the XPS data. The N1s peaks of these compounds are too close to that of N-6 to separate. N-5 species have the highest content in the XPS data, while N-6 is the main nitrogen-containing species and no N-Q is identified in the GC-MS data. N-6 with the highest alkalinity combines with acid sites and is mostly inside the coke on the catalysts. N-Q in the XPS data may actually be the protonated N formed by the combination of N-6 and H+ at the active center of the catalysts. Coke information obtained from surface analysis (XPS) is limited and subject to interference from the catalyst bulk, which is determined with the GC-MS methodology. The explicit molecular structure of the coke inside the spent catalyst can be obtained by GC-MS after dissolution with hydrofluoric acid and extraction with methylene chloride. After the catalyst framework is destroyed, the trapped coke is released to give more insight into the chemical nature of the pollutant precursors.

S3. *In situ* Raman Spectroscopy Results


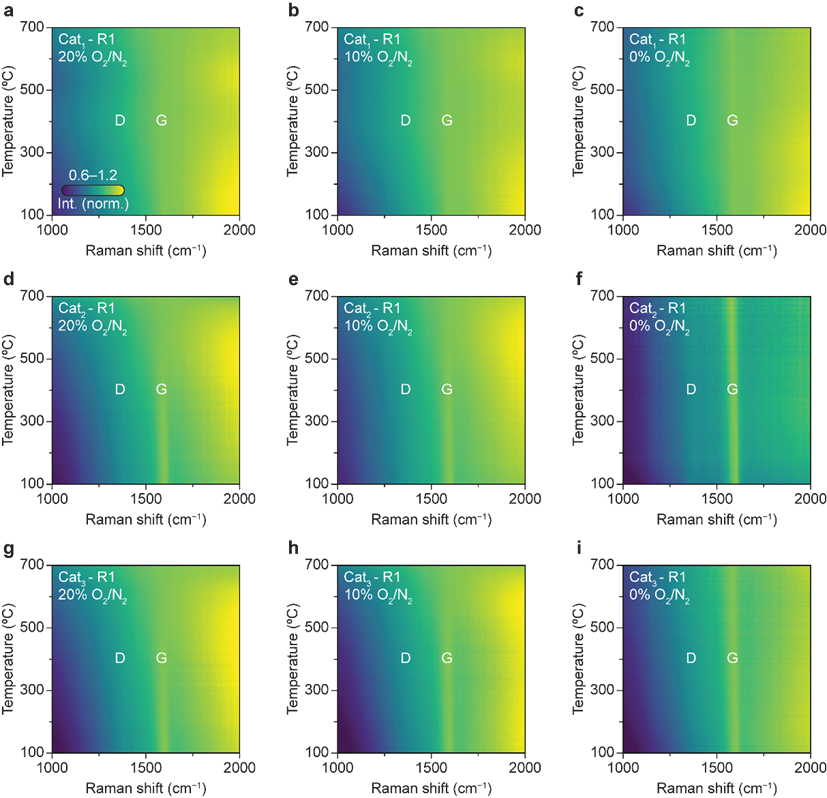


**Figure S4.** Heatmap of the *in-situ* Raman spectroscopy data. **(a)-(c)**, Cat_1_ regeneration in 20%, 10%, and 0% O_2_/N_2_ atmosphere. **(d)-(f)**, Cat_2_ regeneration in 20%, 10%, and 0% O_2_/N_2_ atmosphere. **(g)-(i)**, Cat_3_ regeneration in 20%, 10%, and 0% O_2_/N_2_ atmosphere. At each time, the spectrum is normalized to the maximum of the G-band.


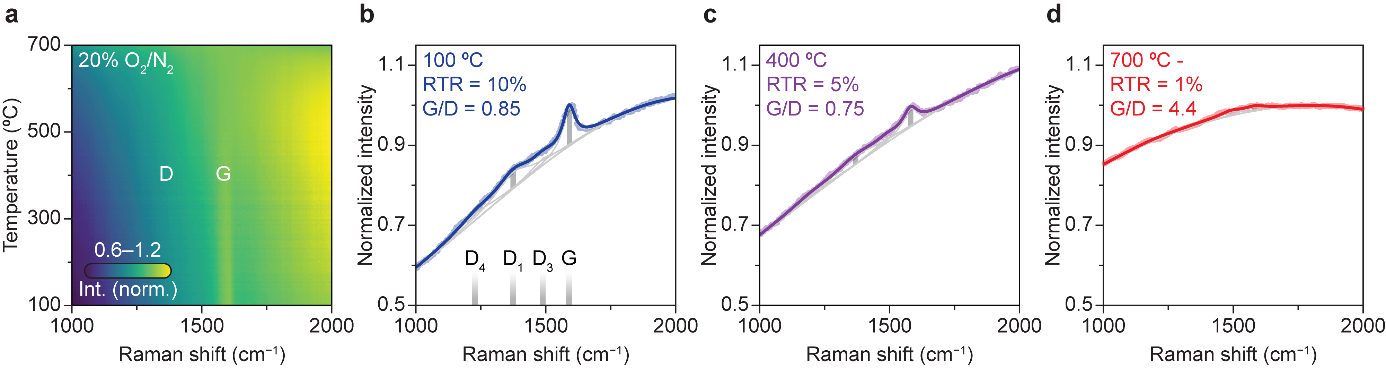


**Figure S5.** Spectral fitting procedure of the Raman spectra recorded during the regeneration of Cat_2_. **(a)** Heatmap of the Raman spectrum recorded during the regeneration of Cat_2_ in 20% O_2_/N_2_. At each time, the spectrum is normalized to the maximum of the G-band. The results of the fitting procedure of the spectrum were recorded at 100 **(b)**, 400 **(c)**, and 700 **(d)** °C. The spectra are normalized to the maximum of the G-band and shown as datapoints, the fit as a solid-colored line, and the contributions of the individual Raman bands and the background as solid grey lines. The Raman-to-Total Ratio (RTR) value is calculated with the background-corrected average intensity of the G-band in a range of 20 wavenumbers centered around the position of the line, as demonstrated with the grey areas. The G- to D-band intensity ratio is determined by the areas of G and D_1_.

The Raman spectra recorded during the regeneration of spent FCC particles show distinct Raman features, together with a broad background fluorescence. The RTR and the G- to D-band intensity ratio used in this work are calculated with a spectral fit. The spectra are modeled as a 3^rd^ degree polynomial function for the background, together with a linear combination of Lorentzian line shapes with center $p_{i}$, width $w_{i}$, and area $h_{i}$ to represent the Raman features:

$$f\left( x \right)= ax^{3}+ bx^{2}+cx+d+ \sum_{i} \frac{h_{i}}{\pi}\frac{0.5 w_{i}}{{(x-p_{i})}^{2}+{(0.5 w_{i})}^{2}}$$

Figure S5 shows the results of the fitting procedure of the Raman spectra presented in this work. The heatmap in Figure S3a illustrates that the Raman features disappear around 500°C. The spectra illustrated in Figure S5b-d are fit to a function with four Lorentzian line shapes. The G-band is due to vibrations of a perfect graphitic lattice. The D_1_- and D_3_-band are due to vibrations of a disordered graphitic lattice, while the D_4_-band is assigned to amorphous carbon. The broad D-bands are barely distinguishable from the background, hampering the accurate determination of the G- to D-band intensity ratio.


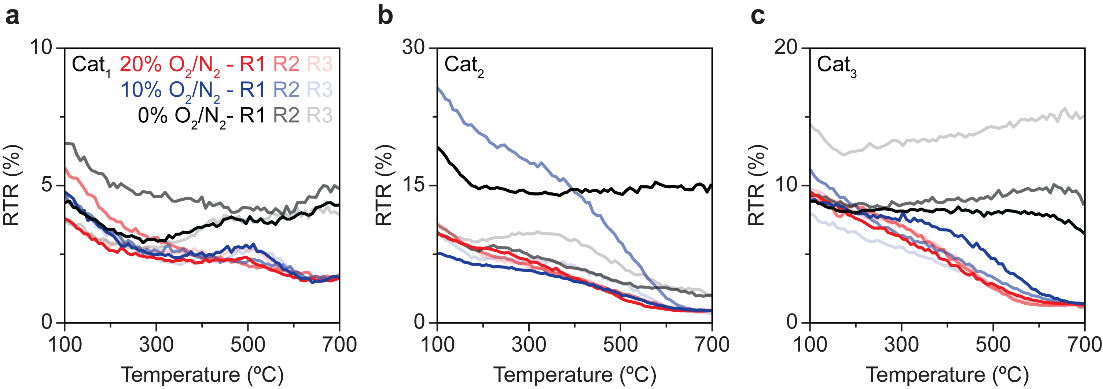


**Figure S6.** Raman-to-Total Ratio (RTR) trends of the G-band as a function of temperature for the regenerations. In 20%, 10%, and 0% O_2_/N_2_ of samples Cat_1_ **(a)**, Cat_2_ **(b)**, and Cat_3_ **(c)**. Each experiment is repeated three times, indicated by the traces of R1, R2, and R3.

Three 10 mg batches of samples Cat_1_, Cat_2_, Cat_3_, were regenerated in 20%, 10% and 0% O_2_/N_2_. *In situ* Raman spectroscopy was used to follow the degree of graphitization of a selected FCC catalyst particle during its regeneration. We determined the RTR trends with a spectral fitting method (Figure S5) and showed the trends for all experiments in Figure S6.

In general, the RTR values of the samples treated in 0% O_2_/N_2_ maintained a relatively constant RTR, indicating that the graphitic structures did not break apart to form fluorescing species. In contrast, samples treated with O_2_ showed clearly decreasing RTR values with increasing temperature. O_2_ facilitates the combustion of larger graphic structures via the formation of smaller fluorescent polyaromatic hydrocarbons. The initial RTR values within one sample vary considerably from particle to particle due to sample heterogeneity.


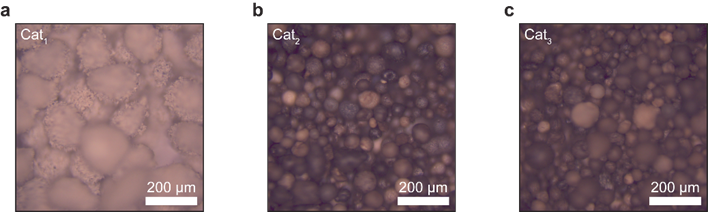


**Figure S7.** Overview of microscopy reflection images of the fluid catalytic cracking (FCC) materials. **(a)**, Cat_1_. **(b)**, Cat_2_. **(c)**, Cat_3_.

The microscopy reflection images of the catalyst materials under study are shown in Figure S7. The FCC particles of Cat_1_, shown in Figure S7a, are relatively large because the clumpy sample was sieved to obtain a fine powder. The particles in Cat_1_ are considerably lighter than the particles within the samples Cat_2_ and Cat_3_, presented in Figure S7 b and c. In addition, these particles vary strongly in shape, size, and color. The sample heterogeneity can be attributed to differences in the age of the catalyst particles under study. A fraction of the FCC particles in an operating FCC unit is replaced with fresh catalyst material at regular intervals. By doing so, the productivity of such a unit is kept stable. As a result, a mixture of FCC particles sampled from an industrial unit is composed of a broad age distribution. The age differences cause variations in catalytic performance and the buildup of coke. Furthermore, FCC particles can be exposed to crude oil fractions containing different amounts of metal poisons (e.g., nickel, iron and vanadium), thereby altering their coking behavior. Hence, the heterogeneity of the FCC samples should be assessed carefully when discussing our Raman microscopy methods, since we probe only one E-cat particle per measurement.


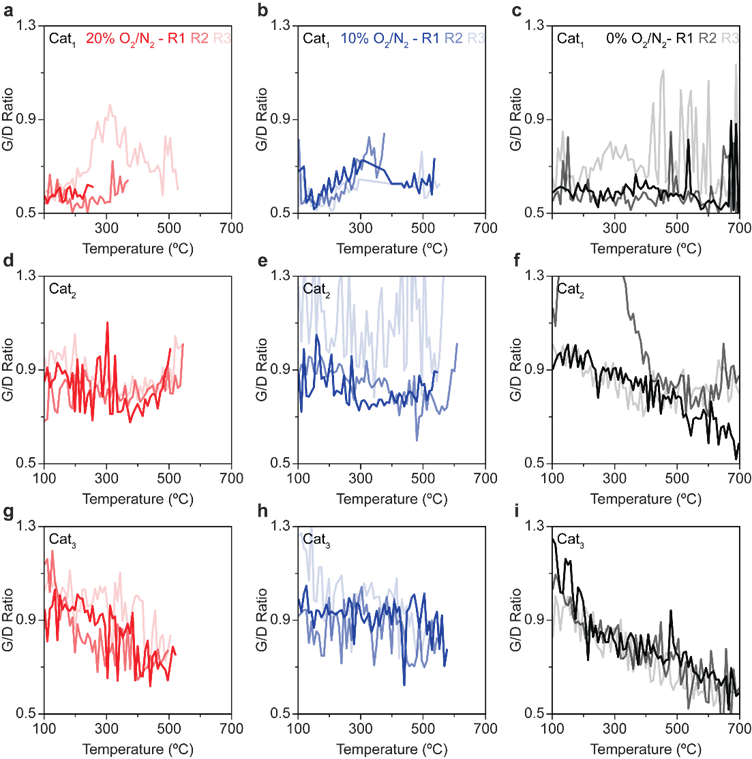


**Figure S8.** G- to D_1_-band intensity ratio (G/D ratio) trends of the G-band as a function of temperature for the regenerations. In 20% (left), 10% (middle), and 0% O_2_/N_2_ (right) of samples Cat_1_ (top), Cat_2_ (middle) and Cat_3_ (bottom). Each experiment is repeated three times, indicated by the traces of R1, R2, and R3. A ratio at a temperature is only added to the trendline if the Raman-to-total ratio (RTR) value of the G-band is larger than 2.5%.

We determined the G- to D_1_-band intensity ratio trends with a spectral fitting method (Figure S5) and showed the trends for all regeneration experiments in Figure S8. In general, the ratio appears to decrease, which holds especially for the data reported in Figure S8g-i. This trend might suggest the shrinking of large well-ordered graphitic structures during the regeneration. This trend however is not reproduced for the other measurements. Furthermore, the noisy trends underline that the D-bands and the background are not well distinguishable. At one temperature, the fitting procedure attributes plenty of the signal to the D_1_-band, to assign most of the signal to the background at the following temperature. This results in large and random fluctuations of the ratio trends, making a chemical interpretation of this data infeasible.

S4. Confocal Fluorescence Microscopy Images


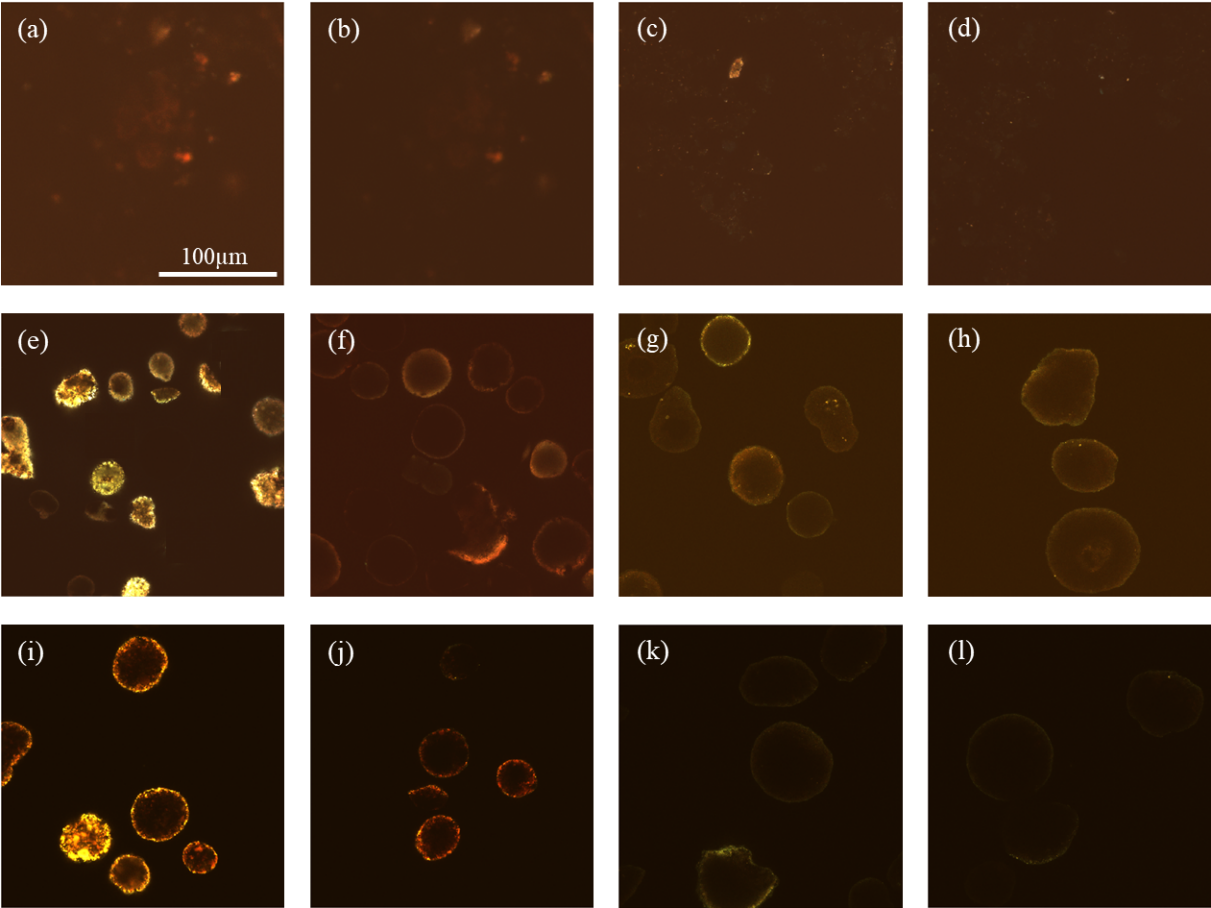


**Figure S9.** Confocal fluorescence microscopy (CFM) images of spent and regenerated fluid catalytic cracking (FCC) materials. **(a)**, Cat_1_. **(b)-(d)**, regenerated Cat_1_ in 0%, 10%, and 20% O_2_/N_2_ atmosphere. **(e)**, Cat_2_. **(f)-(h)**, regenerated Cat_2_ in 0%, 10%, and 20% O_2_/N_2_ atmosphere. **(i)**, Cat_3_. **(j)-(l),** regenerated Cat_3_ in 0%, 10%, and 20% O_2_/N_2_ atmosphere.

S5. Thermogravimetric Analysis Results


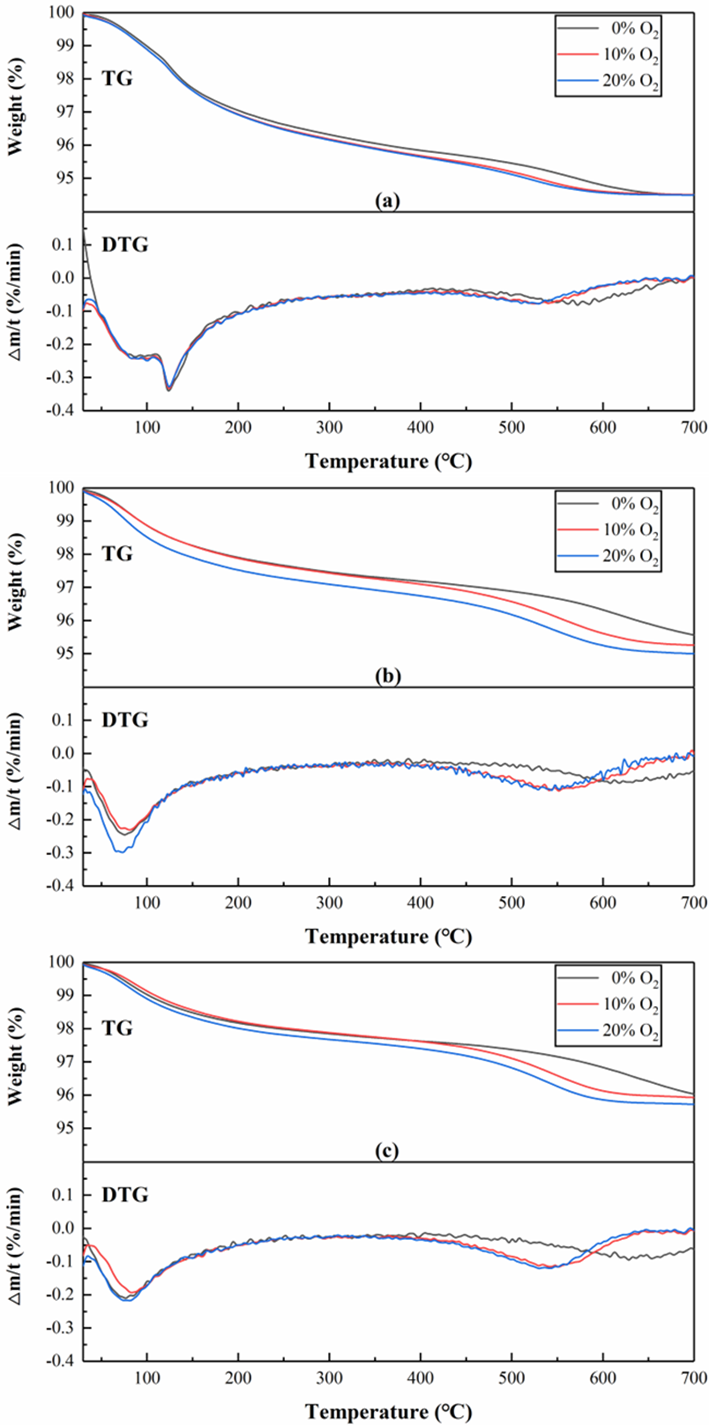


**Figure S10.** Thermogravimetric/differential thermogravimetric (TGA/DTG) analysis curves of spent fluid catalytic cracking (FCC) materials in different atmospheres. **(a)**, Cat_1_. **(b)**, Cat_2_. **(c)**, Cat_3_.

S6. *Operando* Infrared Spectroscopy Results


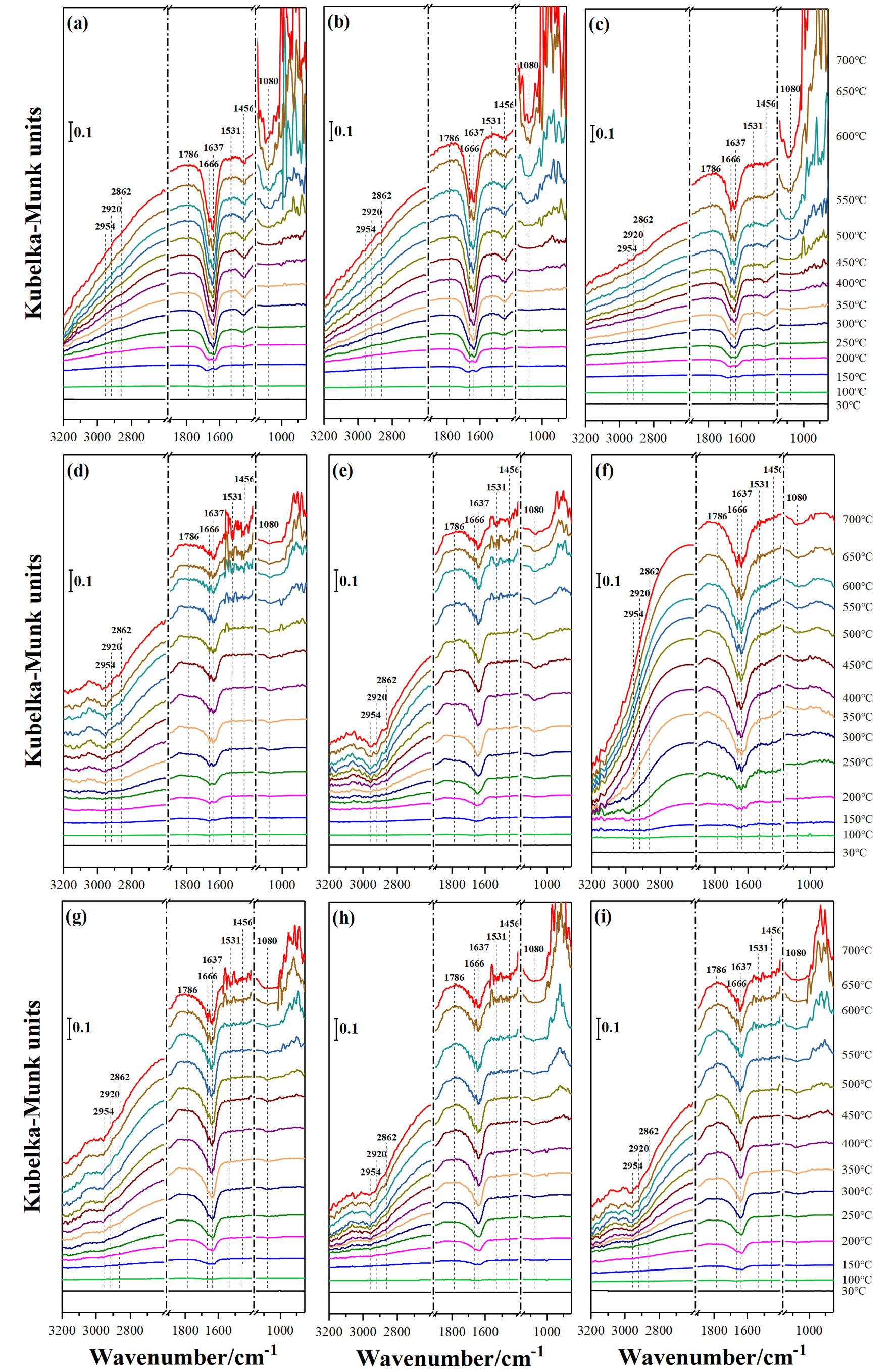


**Figure S11.** *Operando* Fourier transform-infrared (FT-IR) spectroscopy data of spent fluid catalytic cracking (FCC) materials during regeneration. **(a)-(c)**, Cat_1_ regeneration in 0%, 10%, and 20% O_2_/N_2_ atmosphere. **(d)-(f)**, Cat_2_ regeneration in 0%, 10%, and 20% O_2_/N_2_ atmosphere. **(g)-(i)**, Cat_3_ regeneration in 0%, 10%, and 20% O_2_/N_2_ atmosphere.

**Table S9.** Band assignments for functional groups in the in-situ Fourier transform-infrared (FT-IR) spectra.

| **Wavenumber (cm**^−^**^1^)** | **Functional group** | **Assignment** |
| --- | --- | --- |
| 3000-2800 | Aliphatic C-H | Stretching vibration of C-H |
| 1788 | COO- | Stretching vibration of C=O |
| 1666 | C=O | Stretching vibration of C=O |
| 1637 | H_2_O | Bending vibration of O-H |
| 1531 | C=C | Stretching vibration of C=C in aromatic ring |
| 1456 | C-N | Stretching vibration of C-N |
| 1080 | C-O-C | Stretching vibration of C-O |

S7. Online Gas-Phase Infrared Spectroscopy Results


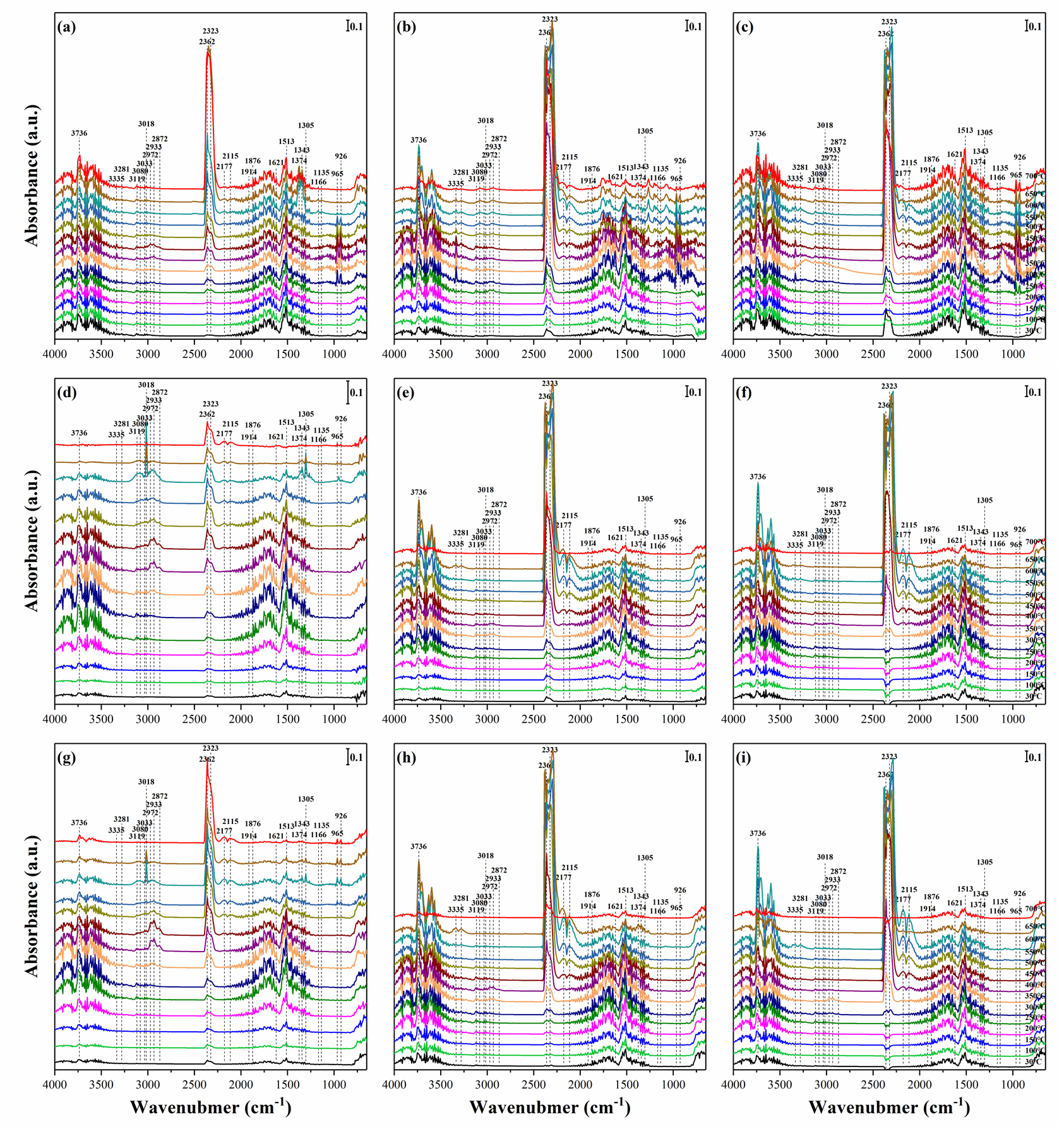


**Figure S12.** Online Fourier transform-infrared (FT-IR) spectroscopy data of gas products formed during catalyst regeneration. **(a)-(c)**, Cat_1_ regeneration in 0%, 10%, and 20% O_2_/N_2_ atmosphere. **(d)-(f)**, Cat_2_ regeneration in 0%, 10%, and 20% O_2_/N_2_ atmosphere. **(g)-(i)**, Cat_3_ regeneration in 0%, 10%, and 20% O_2_/N_2_ atmosphere.

**Table S10.** Infrared (IR) spectral range for vapors and gases during fluid catalytic cracking (FCC) materials regeneration.

| **Wavenumber (cm**^−^**^1^)** | **Gas component** |
| --- | --- |
| 3736, 1513 | H_2_O |
| 3335, 3281 | HCN |
| 3119, 3080, 3033 | Aromatic C-H |
| 3018, 1305 | CH_4_ |
| 2972, 2933, 2872 | Aliphatic C-H |
| 2362, 2323 | CO_2_ |
| 2177, 2115 | CO |
| 1914, 1876 | NO |
| 1621 | NO_2_ |
| 1374, 1343, 1166, 1135 | SO_2_ |
| 965, 962 | NH_3_ |


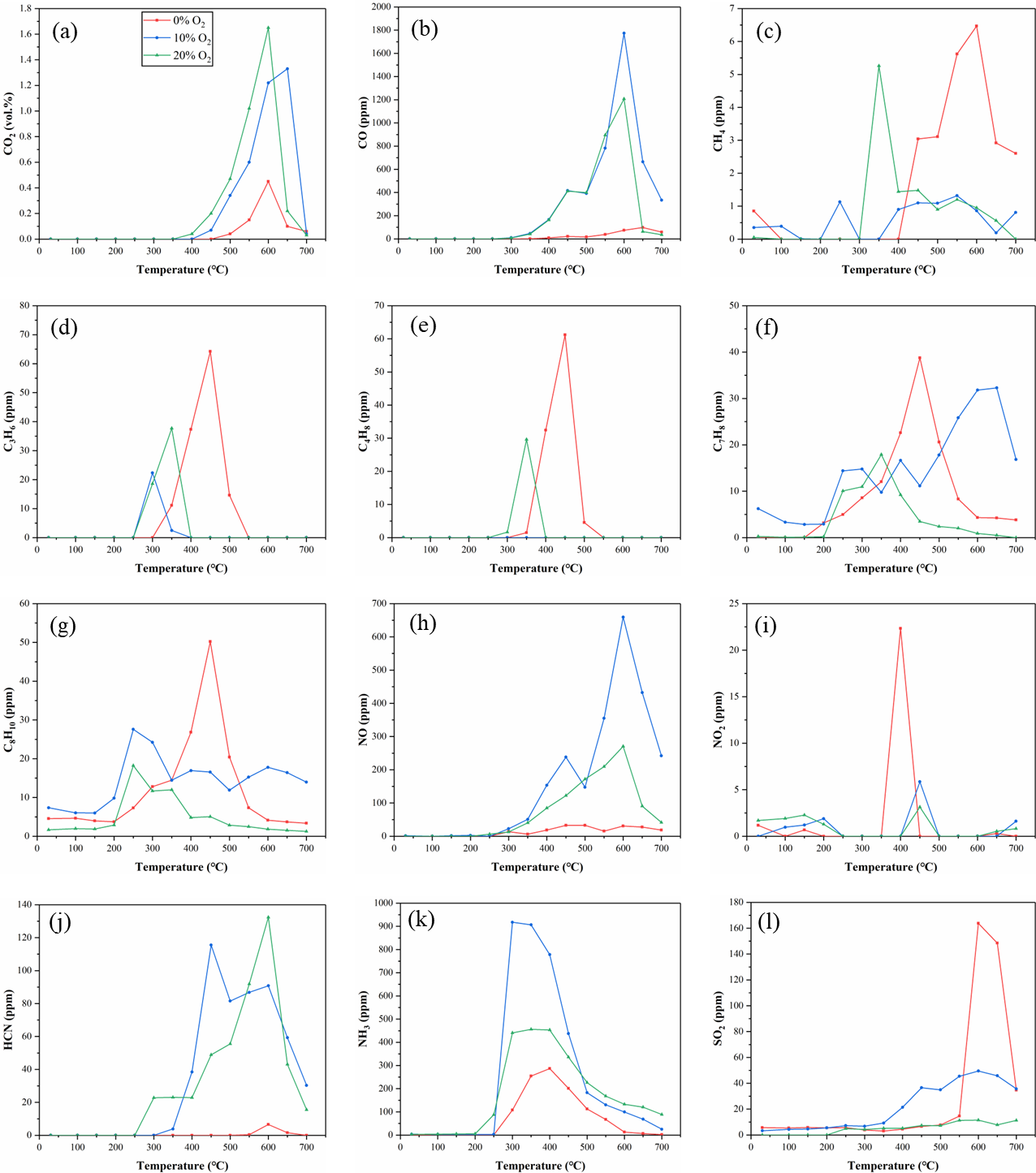


**Figure S13.** Emission concentrations of gas pollutants during Cat_1_ regeneration experiments from online Fourier transform-infrared (FT-IR) spectroscopy data. **(a)**, CO_2_. **(b)**, CO. **(c)**, CH_4_. **(d)**, propylene (C_3_H_6_). **(e)**, butene (C_4_H_8_). **(f)**, toluene (C_7_H_8_). **(g)**, ethylbenzene (C_8_H_10_). **(h)**, NO. **(i)**, NO_2_. (j), HCN. (k), NH_3_. (l), SO_2_.


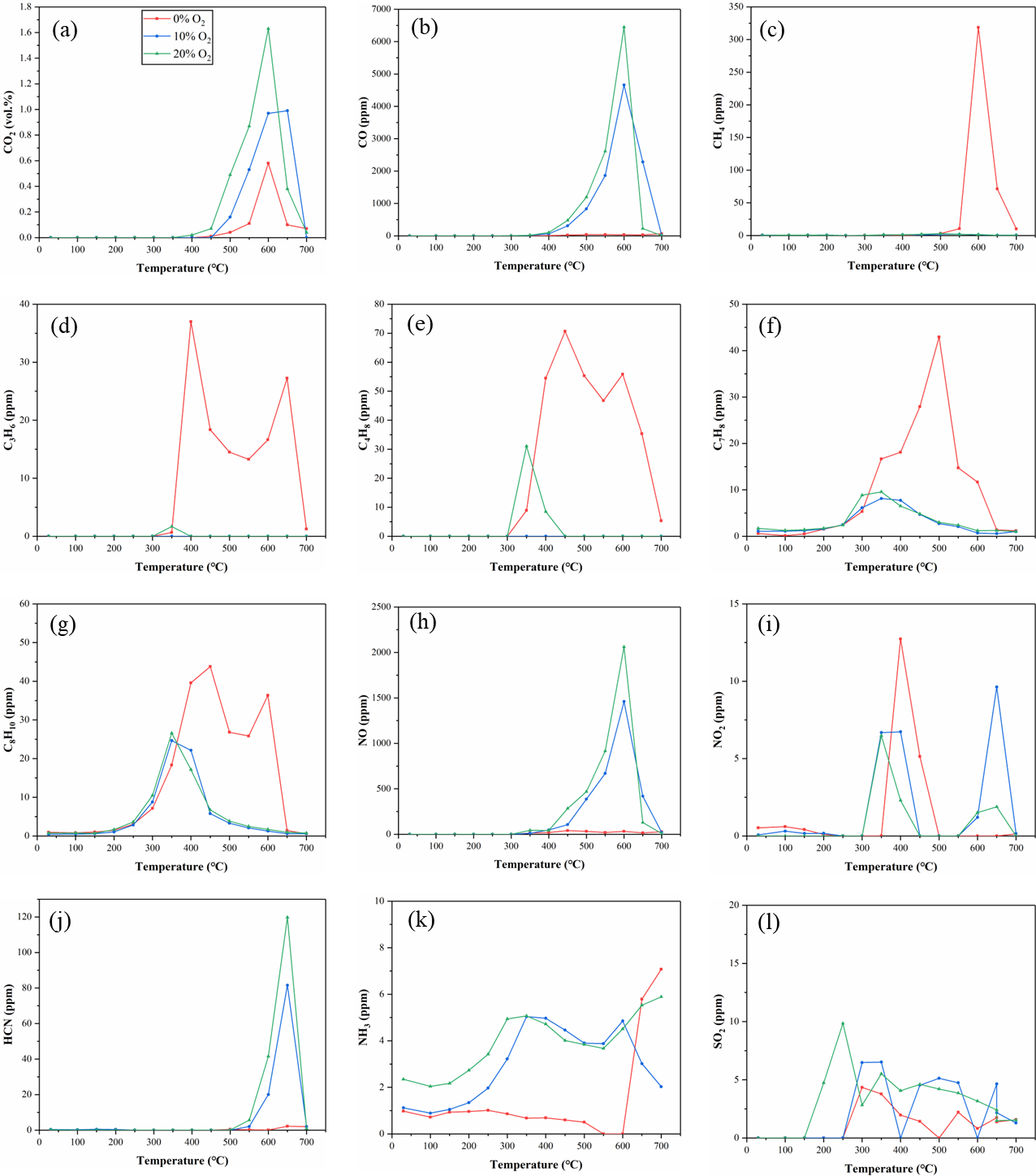


**Figure S14.** Emission concentrations of gas pollutants during Cat_2_ regeneration experiments from online Fourier transform-infrared (FT-IR) spectroscopy data. **(a)**, CO_2_. **(b)**, CO. **(c)**, CH_4_. **(d)**, propylene (C_3_H_6_). **(e)**, butene (C_4_H_8_). **(f)**, toluene (C_7_H_8_). **(g)**, ethylbenzene (C_8_H_10_). **(h)**, NO. **(i)**, NO_2_. (j), HCN. (k), NH_3_. (l), SO_2_.


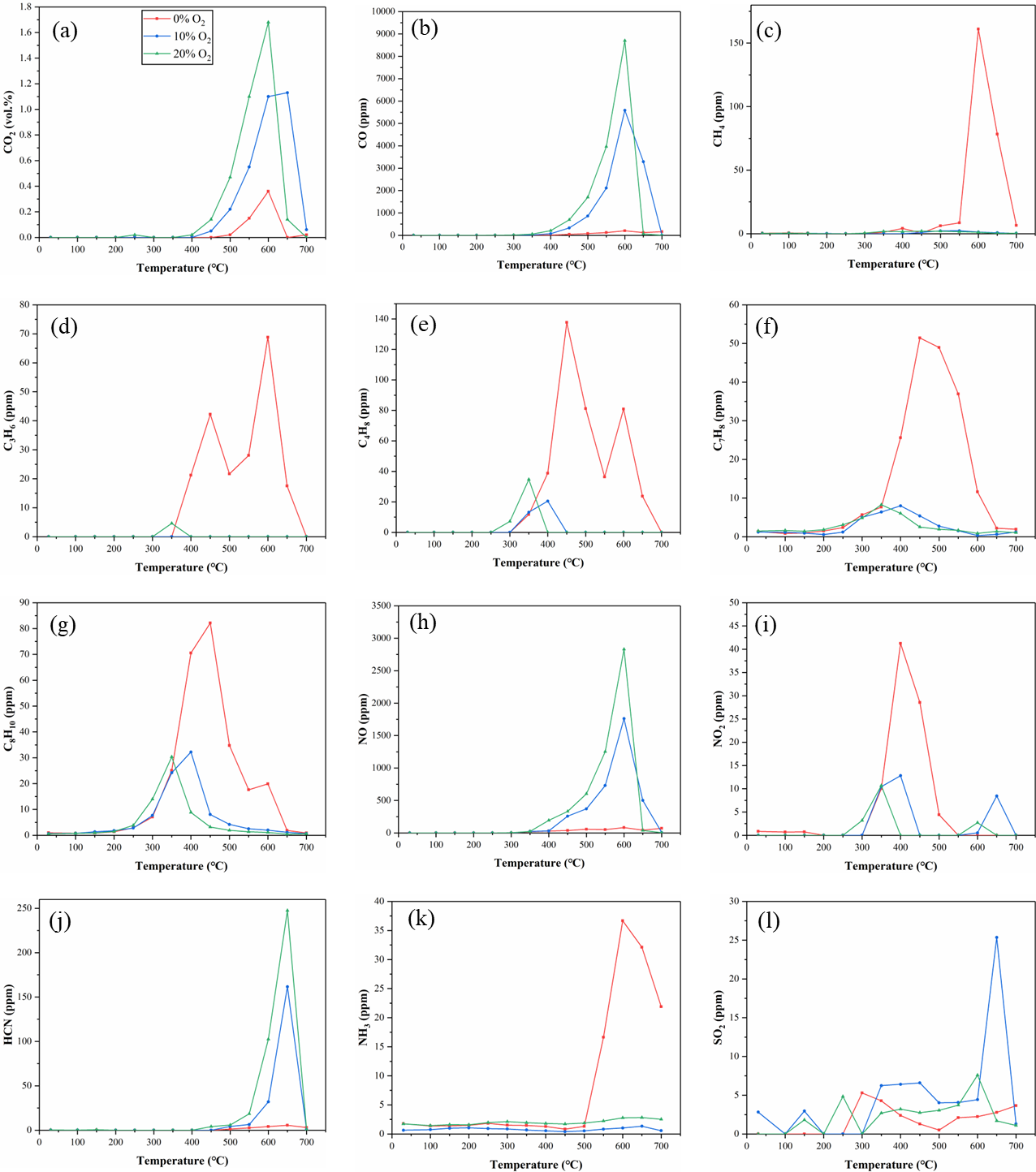


**Figure S15.** Emission concentrations of gas pollutants during Cat_3_ regeneration experiments from online Fourier transform-infrared (FT-IR) spectroscopy data. **(a)**, CO_2_. **(b)**, CO. **(c)**, CH_4_. **(d)**, propylene (C_3_H_6_). **(e)**, butene (C_4_H_8_). **(f)**, toluene (C_7_H_8_). **(g)**, ethylbenzene (C_8_H_10_). **(h)**, NO. **(i)**, NO_2_. (j), HCN. (k), NH_3_. (l), SO_2_.

S8. Density Functional Theory Calculations


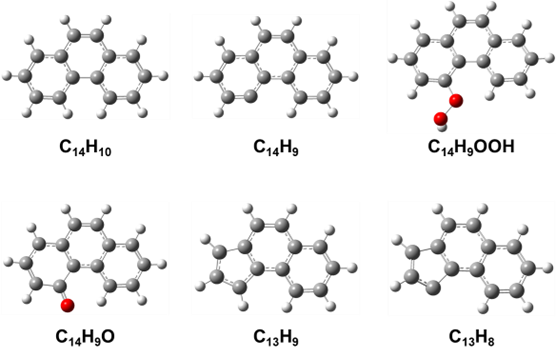


**Figure S16.** Optimized structures of reactants and intermediates for the production of CO and CO_2_. Gray, white, and red spheres represent C, H, and O atoms, respectively.


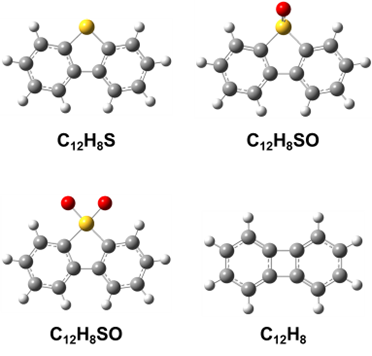


**Figure S17.** Optimized structures of reactants and intermediates for the production of SO_2_. Gray, yellow, white, and red spheres represent C, S, H, and O atoms, respectively.


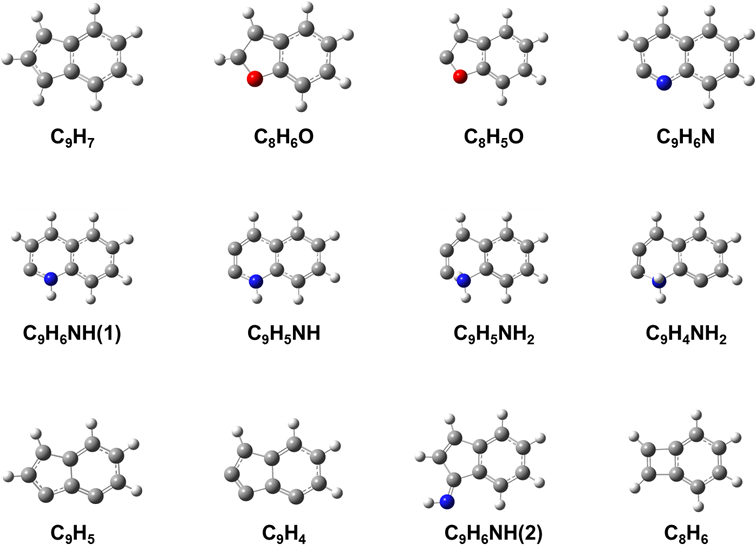


**Figure S18.** Optimized structures of reactants and intermediates for the production of nitrogen compounds, namely NO, NO_2_, NH_3_, and HCN. Gray, blue, white, and red spheres represent C, N, H, and O atoms, respectively.


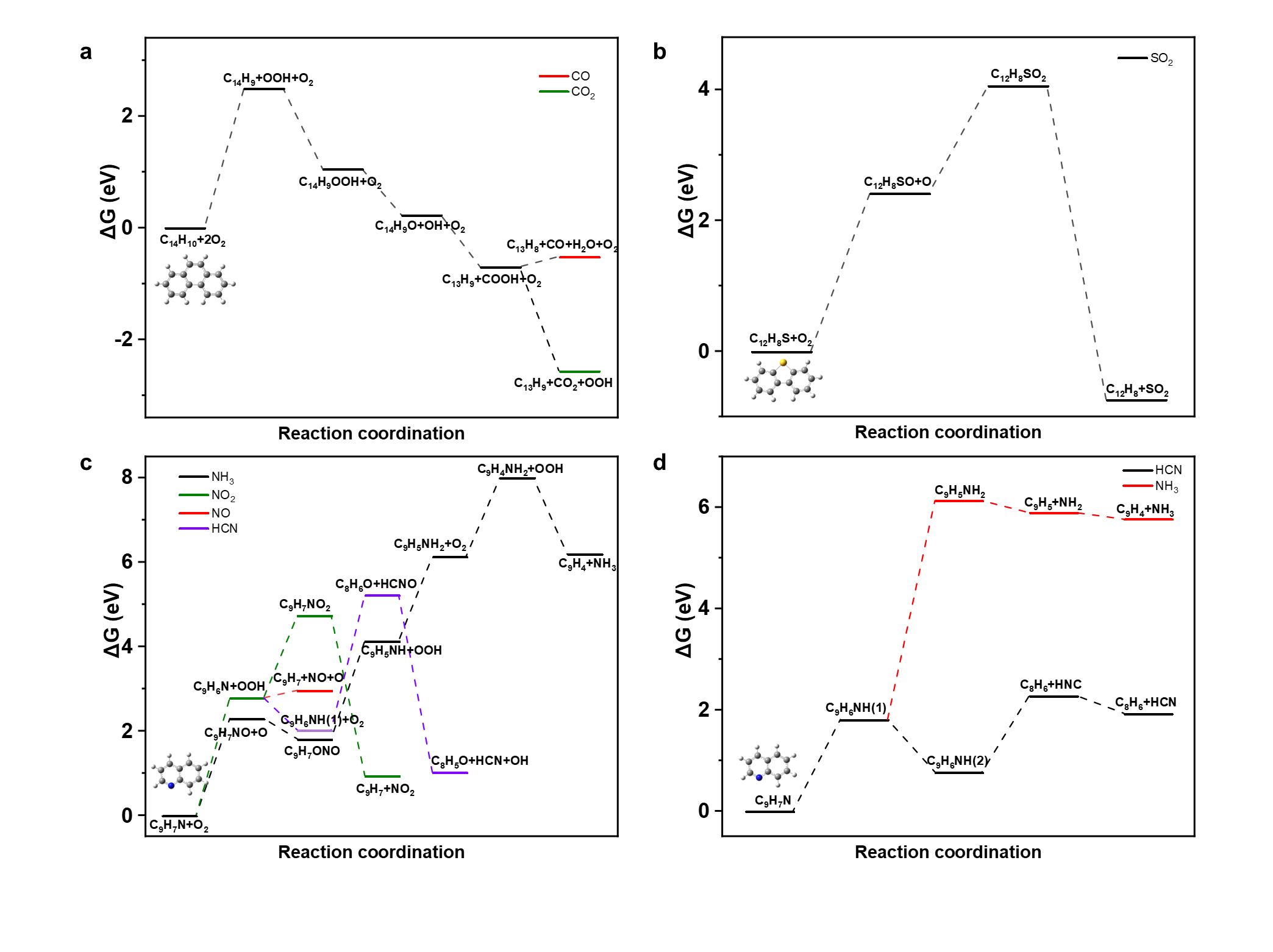


**Figure S19.** DFT calculated free energy (∆G) of the evolution of coke molecules at 700^o^C and 1 atm. **a**, The formation of CO and CO_2_ from C_14_H_10_ in the presence of O_2_. **b**, The formation of SO_2_ from C_12_H_8_S in the presence of O_2_. **c**, The formation of NO, NO_2_, NH_3_ and HCN from C_9_H_7_N in the presence of O_2_. **d**, The formation of NH_3_ and HCN from C_9_H_7_N under oxygen-free conditions. Gray, blue, and white spheres represent C, N, and H atoms, respectively.

S9. Reference

[1] M. J. Frisch, G. W. Trucks, H. B. Schlegel, G. E. Scuseria, M. A. Robb, J. R. Cheeseman, G. Scalmani, V. Barone, B. Mennucci, G. A. Petersson, H. Nakatsuji, M. Caricato, X. Li, H. P. Hratchian, A. F. Izmaylov, J. Bloino, G. Zheng, J. L. Sonnenberg, M. Hada, M. Ehara, K. Toyota, R. Fukuda, J. Hasegawa, M. Ishida, T. Nakajima, Y. Honda, O. Kitao, H. Nakai, T. Vreven, J. A. Montgomery, Jr., J. E. Peralta, F. Ogliaro, M. Bearpark, J. J. Heyd, E. Brothers, K. N. Kudin, V. N. Staroverov, R. Kobayashi, J. Normand, K. Raghavachari, A. Rendell, J. C. Burant, S. S. Iyengar, J. Tomasi, M. Cossi, N. Rega, J. M. Millam, M. Klene, J. E. Knox, J. B. Cross, V. Bakken, C. Adamo, J. Jaramillo, R. Gomperts, R. E. Stratmann, O. Yazyev, A. J. Austin, R. Cammi, C. Pomelli, J. W. Ochterski, R. L. Martin, K. Morokuma, V. G. Zakrzewski, G. A. Voth, P. Salvador, J. J. Dannenberg, S. Dapprich, A. D. Daniels, O. Farkas, J. B. Foresman, J. V. Ortiz, J. Cioslowski, D. J. Fox, *Gaussian 09, Revision C.01*, Gaussian, Inc., Wallingford CT, **2010**.
